# Meiotic chromosome synapsis depends on multivalent SYCE1-SIX6OS1 interactions that are disrupted in cases of human infertility

**DOI:** 10.1126/sciadv.abb1660

**Published:** 2020-09-02

**Authors:** Fernando Sánchez-Sáez, Laura Gómez-H, Orla M. Dunne, Cristina Gallego-Páramo, Natalia Felipe-Medina, Manuel Sánchez-Martín, Elena Llano, Alberto M. Pendas, Owen R. Davies

**Affiliations:** 1 Molecular Mechanisms Program, Centro de Investigación del Cáncer and Instituto de Biología Molecular y Celular del Cáncer (CSIC-Universidad de Salamanca), Salamanca, Spain.; 2 Biosciences Institute, Faculty of Medical Sciences, Newcastle University, Framlington Place, Newcastle upon Tyne NE2 4HH, UK.; 3 Departamento de Medicina, Universidad de Salamanca, Salamanca, Spain.; 4 Departamento de Fisiología y Farmacología, Universidad de Salamanca, Salamanca, Spain.

## Abstract

Meiotic reductional division depends on the synaptonemal complex (SC), a supramolecular protein assembly that mediates homologous chromosomes synapsis and promotes crossover formation. The mammalian SC has eight structural components, including SYCE1, the only central element protein with known causative mutations in human infertility. We combine mouse genetics, cellular, and biochemical studies to reveal that SYCE1 undergoes multivalent interactions with SC component SIX6OS1. The N terminus of SIX6OS1 binds and disrupts SYCE1’s core dimeric structure to form a 1:1 complex, while their downstream sequences provide a distinct second interface. These interfaces are separately disrupted by SYCE1 mutations associated with nonobstructive azoospermia and premature ovarian failure (POF), respectively. Mice harboring SYCE1’s POF mutation and a targeted deletion within SIX6OS1’s N terminus are infertile with failure of chromosome synapsis. We conclude that both SYCE1-SIX6OS1 binding interfaces are essential for SC assembly, thus explaining how SYCE1’s reported clinical mutations give rise to human infertility.

## Teaser

Synaptonemal complex assembly depends on multivalent protein interactions between SYCE1 and SIX6OS1.

## INTRODUCTION

Meiotic cell division is defined by a unique and highly dynamic program of events that result in homologous chromosome synapsis, crossover (CO) formation, and subsequent homolog segregation into haploid germ cells (*1*–*3*). Homologous chromosome pairs are established through interhomolog recombination searches from up to 400 induced double-strand breaks (DSBs) per cell (*4*). Once established, local recombination-mediated alignments are converted into the single continuous synapsis of aligned homologous chromosomes through the zipper-like assembly of the synaptonemal complex (SC) (*5*). The SC’s supramolecular protein structure mediates continuous 100-nm tethering between homologous chromosome axes and provides the necessary three-dimensional framework for crossover formation (*2*). Following SC disassembly, crossovers provide the sole physical links between homologs at metaphase I, so are essential for ensuring correct homolog segregation in addition to providing genetic diversity (*2*).

**Fig. 1:**
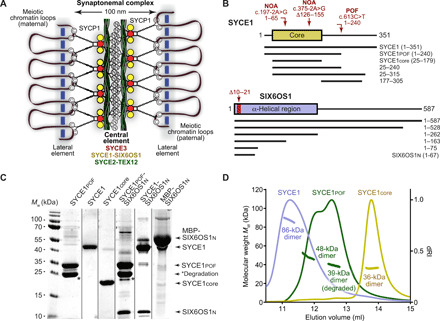
SYCE1_POF_ retains its core dimeric structure. (**A**) Schematic of the SC demonstrating its tripartite structure of two chromosome-bound LEs and a midline CE. Synapsis is achieved through N-terminal head-to-head assembly of SYCP1 molecules, which are bound via their C termini to meiotic chromosomes. SYCP1 head-to-head assembly is structurally supported within the CE by SYCE3 (red), an SYCE1-SIX6OS1 complex (yellow), and SYCE2-TEX12 fibrous assemblies (green). (**B**) Human SYCE1 (top) and SIX6OS1 (bottom) sequence schematics indicating the location and consequence of infertility-associated mutations of SYCE1 and Δ10–21 internal deletion of SIX6OS1, alongside the principal constructs used in this study. (**C**) SDS–polyacrylamide gel electrophoresis (SDS-PAGE) analysis of the purified recombinant proteins used in this study. The dominant degradation product of SYCE1_POF_ is indicated by an asterisk; its identity was confirmed by the observed cleavage of degraded MBP- and His-SYCE1_POF_ fusion proteins upon treatment with TEV protease (fig. S1, A and B), consistent with it representing C-terminal degradation down to SYCE1’s structural core. *M*_w_, weight-average molecular weight. (**D**) SEC-MALS analysis. SYCE1core (yellow), SYCE1_POF_ (green), and full-length SYCE1 (violet) are dimeric species of 36, 48 (39 kDa for the degradation product), and 86 kDa, respectively (theoretical dimers: 37, 55, and 80 kDa). dRI, differential refractive index. Data for SYCE1core and full-length SYCE1 are reproduced from (*28*).

The SC has an iconic and highly conserved tripartite structure that has been observed across meiotically reproducing eukaryotes (*6*). This consists of lateral elements (LEs) that coat the two homologous chromosome axes and a midline central element (CE), with a series of transverse filaments that bind together these longitudinal electron-dense structures (Fig. 1A) (*7*). The protein components of the mammalian SC have been identified as transverse filaments protein SYCP1 (Synaptonemal complex protein 1) (*8*), CE proteins SYCE1, SYCE2, and SYCE3 (Synaptonemal complex central element proteins 1 to 3), SIX6OS1, and TEX12 (Testis-expressed protein 12) (*9*–*12*), and LE proteins SYCP2 and SYCP3 (*13*, *14*). All transverse filament and CE components are essential for SC assembly, and their individual disruption leads to infertility owing to meiotic arrest with failure of DSB repair (*10*, *11*, *15*–*18*). In contrast, disruption of LE components produces a sexual dimorphism of male infertility and female subfertility (*19*, *20*), with SYCP3 deficiency in females promoting germ cell aneuploidy and embryonic death (*21*).

In recent years, a variety of cellular imaging, biochemical and structural biology approaches have begun to uncover the molecular structures, interactions, and mechanisms responsible for mammalian SC assembly. SYCP1 self-assembles into a supramolecular lattice that provides the underlying 100-nm synapsis between chromosome axes (*22*, *23*), while SYCP3 assembles into regularly repeating filaments that support chromosomal looping (*24*, *25*). The five CE proteins provide essential structural supports for the SYCP1 lattice that enable its continuous and cooperative extension along the entire chromosome length. In this capacity, CE proteins have been categorized as synaptic initiation factors (SYCE3, SYCE1, and SIX6OS1) and elongation factors (SYCE2 and TEX12), of which their disruption leads to complete loss of tripartite SC structure and failure of extension of short SC-like stretches, respectively (*10*, *11*, *16*–*18*). Of synaptic initiation factors, SYCE3 forms dimers that undergo potentially limitless self-assembly (*26*, *27*), SYCE1 forms antiparallel dimeric assemblies (*28*), and SIX6OS1 is an SYCE1-interacting protein of unknown structure (*11*). These likely act as short-range structural supports between SYCP1 molecules, possibly in transverse, longitudinal, and vertical orientations to stabilize a local three-dimensional SYCP1 lattice (*22*). In contrast, SYCE2 and TEX12 exist as a seemingly constitutive complex that undergoes self-assembly into fibers of many micrometers in length (*29*), which likely provide the long-range structural supports that stabilize continuous growth of the SYCP1 lattice along the entire chromosome axis (*22*).

Owing to the essential roles of meiotic recombination, synapsis, and chromosome dynamics in mammalian meiosis (*15*, *30*–*34*), their defects are associated with human infertility, recurrent miscarriage, and aneuploidies (*35*, *36*). As genetic causes of infertility, they typically fall within the category of idiopathic cases, having no readily diagnosable and clinically resolvable cause. Within the 10 to 15% of couples who suffer from infertility, approximately 25% are idiopathic and of likely genetic origin, comprising 50 to 80% of cases of nonobstructive azoospermia (NOA) and premature ovarian failure (POF) (*36*, *37*). While individual infertility mutations are inherently unlikely to become widespread in a population, they can be found within families, especially when consanguineous (*38*), and provide crucial insights into their common targets and the molecular mechanisms that they disrupt.

Within the SC, familial infertility mutations have been identified for SYCP3 and SYCE1 (*36*). All identified SYCP3 mutations are autosomal dominant and alter or delete its structural core’s C terminus that mediates filamentous assembly, so likely sequester wild-type (WT) molecules into inactive complexes (*24*, *36*). In contrast, the three identified SYCE1 mutations are autosomal recessive and were found in two familial cases of NOA and one of POF (*36*). The two NOA cases are splice-site mutations, c.197-2A>G and c.375-2A>G, which are predicted to result in a truncated product of amino acids 1 to 65 and an internal deletion of amino acids 126 to 155, respectively (*39*, *40*). These remove or delete part of human SYCE1’s structural core that is encoded by amino acids 25 to 179, so can be explained by disruption of its dimeric structure (Fig. 1B) (*28*, *36*). The POF mutation c.613C>T generates a premature stop codon (p.Gln241*) to give a truncated product of amino acids 1 to 240, relative to the canonical 351–amino acid isoform (Fig. 1B) (*41*). However, as this truncation lies outside SYCE1’s structural core, the molecular mechanism that is disrupted, and thereby responsible for infertility, remains unknown.

Here, we combine mouse genetics and cellular and biochemical studies to reveal a multivalent interaction mode between SYCE1 and SIX6OS1 that is disrupted by infertility-associated mutations of SYCE1. We find that the SIX6OS1 N terminus binds and disrupts the core dimeric structure of SYCE1 (amino acids 25 to 179) to form a 1:1 complex as the first interface, and its downstream sequence binds to SYCE1 amino acids 177 to 305 as the second interface. SYCE1’s infertility-associated mutations c.375-2A>G (NOA) and c.613C>T (POF) specifically disrupt the first and second interfaces, respectively. Mice harboring the SYCE1 POF mutation and a targeted deletion within SIX6OS1 (which disrupts the first interface) are infertile, with failure of SC assembly. We conclude that both SYCE1-SIX6OS1 binding interfaces are essential for SC assembly and meiotic division, thus explaining how human infertility results from the differential targeting of binding interfaces by SYCE1’s reported clinical mutations.

## RESULTS

### SYCE1 POF mutation c.613C>T retains its core dimeric structure

The SYCE1 POF mutation c.613C>T encodes a premature stop codon (p.Gln241*) that is predicted to generate a truncated protein product of amino acids 1 to 240, relative to SYCE1’s canonical 351–amino acid isoform (Fig. 1B) (*41*). We previously demonstrated that an N-terminal structural core encoded by amino acids 25 to 179 (SYCE1core) forms an α-helical antiparallel coiled-coil structure that mediates head-of-head dimerization of SYCE1 (*28*). As this core region is retained (Fig. 1B), we predicted that SYCE1’s antiparallel dimeric structure would be maintained within the 1- to 240-amino acid truncated product of the POF mutation (SYCE1pof). To test this, we purified recombinant SYCE1pof, generating purified material that contained approximately equal quantities of the full protein and a degradation product of apparent size consistent with degradation to the C-terminal boundary of its structural core (Fig. 1C and fig. S1, A and B). Circular dichroism (CD) spectroscopy confirmed that SYCE1pof contains a proportion of α-helical structure consistent with retention of the 25–179 core structure (fig. S1C), and SYCE1pof and SYCE1core demonstrated identical melting temperatures (*T*_m_) of 39°C (fig. S1D). Furthermore, analysis by size exclusion chromatography multiangle light scattering (SEC-MALS) confirmed that the full and degraded proteins are homodimers of 48 and 39 kDa, respectively (Fig. 1D). We conclude that SYCE1pof retains the dimeric structure imposed by its core 25–179 region, so its SC and meiotic defects must result from additional structural or functional roles of its deleted C terminus.

### The SYCE1 POF mutation leads to failure of SC assembly and infertility in mice

**Fig. 2:**
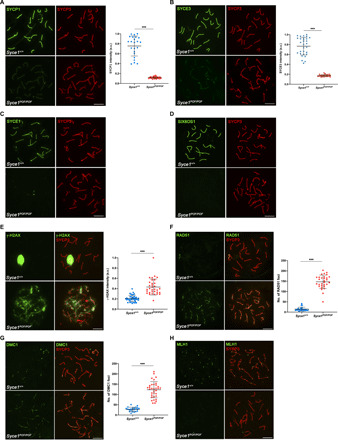
*Syce1^POF/POF^* spermatocytes are not able to synapse and DSBs are deficiently repaired. (**A**) Double immunolabeling of WT pachytene and *Syce1^POF/POF^* zygotene-like spermatocytes with SYCP3 (red) and SYCP1 (green). In *Syce1^POF/POF^* spermatocytes, AEs fail to synapse and show a weak staining of SYCP1 along the axial elements (AEs). a.u., arbitrary units. (**B** to **D**) Double immunolabeling of spermatocyte spreads with SYCP3 (red) and the CE proteins (green). *Syce1^POF/POF^* zygotene-like spermatocytes showed a highly reduced signal of SYCE3 (B) and the absence of (C) SYCE1 and (D) SIX6OS1 from the AEs. (**E**) Double immunolabeling of γ-H2AX (green) and SYCP3 (red) in spermatocyte spreads from WT and *Syce1^POF/POF^* mice. γ-H2AX staining was persistent in *Syce1^POF/POF^* zygotene-like spermatocytes, but was restricted to the sex body in WT pachytene cells. (**F** and **G**) Double immunofluorescence of (F) RAD51 or (G) DMC1 (green) and SYCP3 (red). *Syce1^POF/POF^* zygotene-like spermatocytes showed increased numbers of foci of RAD51 and DMC1 along the AEs in comparison with WT, indicating unrepaired DSBs. (**H**) Double immunolabeling of MLH1 (green) and SYCP3 (red) showing the absence of COs (MLH1) in arrested *Syce1^POF/POF^* spermatocytes. Fluorescence intensity levels (A, B, and E) and number of foci (F and G) from WT and zygotene-like arrested spermatocytes are quantified in the right-hand plots. Welch’s *t* test analysis: ***P < 0.0001. Scale bars, 10 μm.

**Fig. 3:**
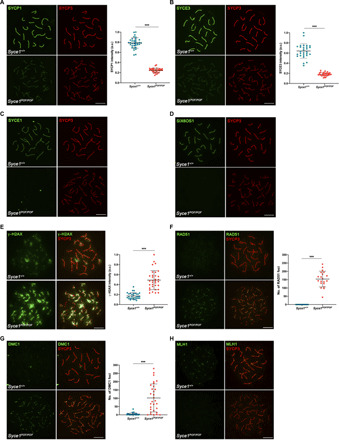
*Syce1^POF/POF^* oocytes fail to synapse and do not properly repair DSBs. (**A**) Double immunolabeling of oocyte spreads from WT and *Syce1^POF/POF^* mice with SYCP3 (red) and SYCP1 (green). *Syce1^POF/POF^* oocytes became arrested in a zygotene-like stage where AEs remain unsynapsed and unaligned, with reduced levels of SYCP1. (**B** to **D**) Double immunolabeling of oocyte spreads with SYCP3 (red) and the CE proteins (green). *Syce1^POF/POF^* zygotene-like oocytes showed reduced SYCE3 signal (B) and a complete absence of (C) SYCE1 and (D) SIX6OS1 from the AEs. IP, immunoprecipitation. (**E**) Double immunostaining of spread preparations of WT pachytene and *Syce1^POF/POF^* zygotene-like oocytes with γ-H2AX (green) and SYCP3 (red). In *Syce1^POF/POF^* oocytes, the levels of γ-H2AX increased and were more restricted to AEs in comparison with WT pachytene cells. (**F** to **G**) Double immunolabeling of (F) RAD51 or (G) DMC1 (green) and SYCP3 (red), showing higher numbers of foci in AEs from mutant oocytes. (**H**) Labeling of MLH1 (green) and SYCP3 (red). MLH1 foci are absent from the AEs of *Syce1^POF/POF^* oocytes. Fluorescence intensity levels (A, B, and E) and number of foci (F and G) from WT and *Syce1^POF/POF^* zygotene-like oocytes are quantified in the right-hand plots. Welch’s *t* test analysis: ***P < 0.0001. Scale bars, 10 μm.

Having established its retention of core dimeric structure, we next sought to determine the structural and functional consequence of the SYCE1 POF mutation on the SC and meiotic division in vivo. We thus generated mice harboring mutations of *Syce1* alleles to introduce stop codons at amino acid position 243, equivalent to the human p.Gln241* mutation (figs. S2 and S3). While heterozygotes (designated *Syce1^POF/WT^*) were fertile, both male and female homozygotes (designated *Syce1^POF/POF^*) were infertile, replicating the autosomal recessive pattern of the POF mutation in humans (*41*). In male mutant mice, we observed reduced testis size (63% smaller, *n* = 3 mice at 2 months of age; fig. S4A) and a zygotene-like arrest similar to that observed in the SYCE1 knockout (*16*). There was defective SC assembly, with reduced staining for SYCP1 (Fig. 2A) and SYCE3 (Fig. 2B) and no staining for SYCE1 (Fig. 2C), SIX6OS1 (Fig. 2D), and SYCE2-TEX12 (fig. S4, B and C). Analysis of SYCE1 expression in the testis of *Syce1^POF/POF^* mice confirmed the presence of *Syce1* transcript and a protein product of the correct molecular weight, albeit at reduced levels in comparison with WT (fig. S4, D and E, and table S1A). The *Syce1^POF^* open reading frame achieved WT levels of protein expression in a heterologous 293T cellular system (fig. S4F). We next studied the kinetics of DSB repair. Meiotic DSBs are generated by the nuclease SPO11 and are then resected to form single-stranded DNA ends that invade into the homologous chromosome by the recombinases RAD51 (DNA repair protein RAD51 homolog 1) and DMC1 (Meiotic recombination protein DMC1/LIM15 homolog) (*42*). DSBs are labeled by the presence of phosphorylated H2AX (γ-H2AX) (*43*). The distribution of γ-H2AX in mutant spermatocytes was similar to that found in WT cells at early prophase I but show increased staining at zygotene-like arrest (Fig. 2E). The distributions of RAD51 and DMC1 were detected on aligned LEs (Fig. 2, F and G) but in absence of mismatch repair protein MLH1 (DNA mismatch repair protein Mlh1) (marker of crossing-overs) (Fig. 2H). Together, these data indicate generation of DSBs but with failure of their repair and CO formation in *Syce1^POF/POF^*. In female mutant mice, we observed no follicles in adult ovaries (fig. S5A), and embryonic oocytes demonstrated zygotene arrest with mostly unaligned chromosome axes, recapitulating the human POF syndrome. Analysis of the SC revealed similar defects, with reduction in SYCP1 and SYCE3 (Fig. 3, A and B) staining (though to a lesser extent than males), and absence of SYCE1, SIX6OS1 (Fig. 3, C and D), and SYCE2-TEX12 (fig. S5, B and C). The distribution of γ-H2AX, RAD51, and DMC1 labeling in zygotene-like mutant oocytes was also increased and lacked MLH1 foci (Fig. 3, E to H). Thus, the SYCE1 POF mutation leads to male and female infertility with phenotypes of failed DSB repair, synapsis, and lastly SC assembly, similar to those previously observed upon disruption of structural components of the SC CE (*10*, *11*, *16*–*18*).

### SYCE1_POF_ retains SIX6OS1 binding but lacks SYCE3 binding in heterologous systems

**Fig. 4:**
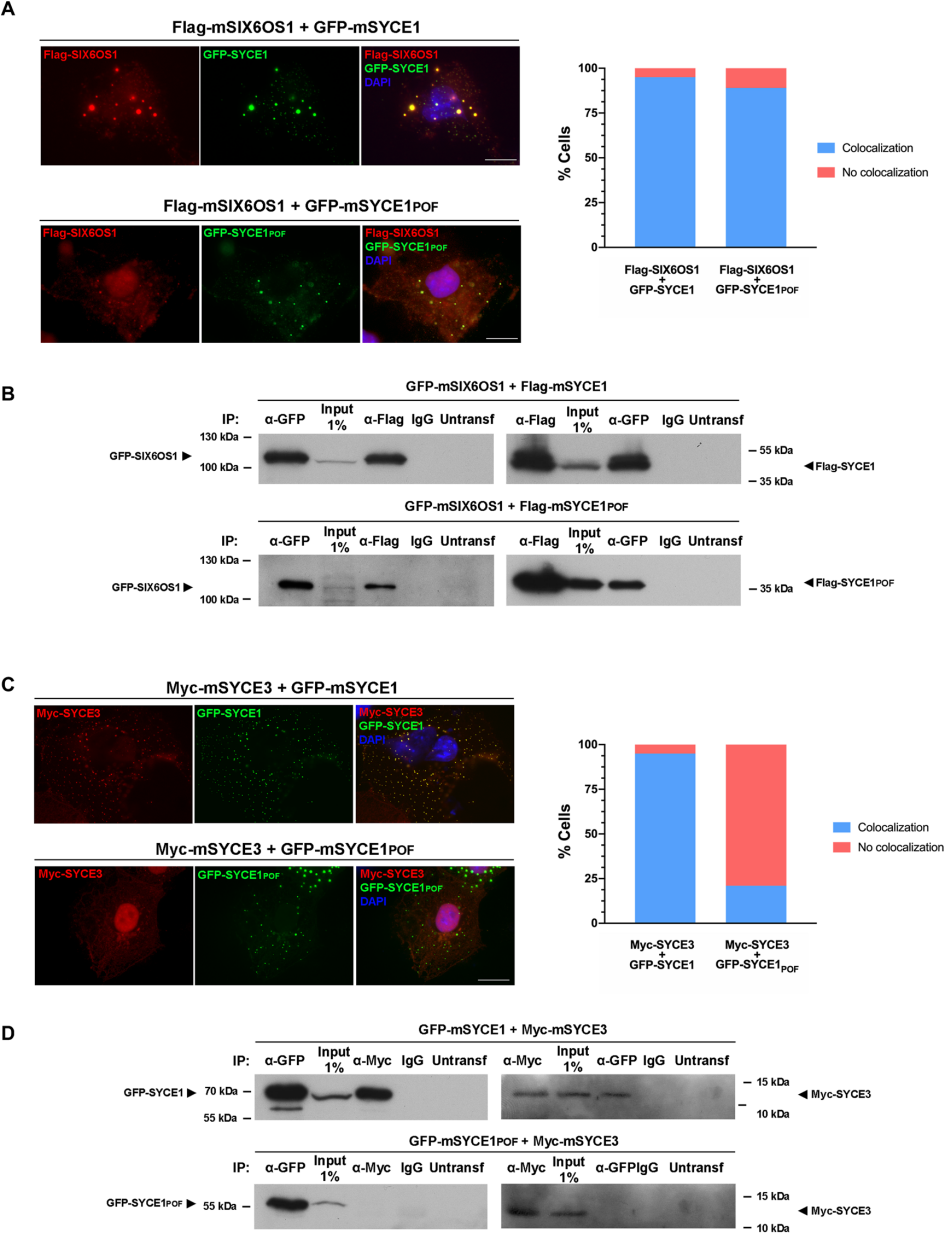
SYCE1_POF_ retains SIX6OS1 binding but fails to retain the SYCE3-interaction in heterologous systems. (**A**) Mouse SIX6OS1 colocalized with mouse SYCE1 and SYCE1_POF_ in a cytoplasmatic punctate pattern upon coexpression in COS7 cells; the percentage of cells exhibiting colocalization is shown in the right-hand plot (*n* = 100 cells). DAPI, 4′,6-diamidino-2-phenylindole. (**B**) HEK293T cells were cotransfected with the indicated expression vectors. Protein complexes were immunoprecipitated with anti-Flag or anti–enhanced green fluorescent protein (EGFP) antibodies, or mouse immunoglobulin G (IgG) as a negative control, and were analyzed by immunoblotting with the indicated antibody. GFP-mSIX6OS1 coimmunoprecipitated with Flag-mSYCE1 and Flag-mSYCE1_POF_, suggesting that the POF mutation of SYCE1 alone is insufficient to block the interaction. (**C**) COS7 cells were transfected with mouse *Syce3* in combination with mouse *Syce1* or *Syce1pof* as indicated. SYCE1 colocalized with SYCE3 in its own cytoplasmatic punctate pattern, and colocalization was substantially diminished for SYCE1_POF_ (*n* = 100 cells). (**D**) Immunoprecipitation of protein complexes from HEK293T-cotransfected cells with an anti-Myc or anti-EGFP antibody or mouse IgG. SYCE1 coimmunoprecipitated with SYCE3, and the interaction was disrupted for SYCE1 POF, suggesting that the C-terminal region of SYCE1 is required for its interaction with SYCE3. The untransfected lanes in (B) and (D) show the absence of all the proteins in total protein extracts from untransfected 293T cells. Scale bars, 20 μm.

As the *Syce1^POF/POF^* mouse strain indicated a clear structural defect in the SC, we wondered whether the POF mutation may disrupt the known interaction between SYCE1 and fellow SC CE components SIX6OS1 and SYCE3 (*11*). The expression of SYCE1 and SIX6OS1 in COS7 cells produced cytoplasmic signals that became colocalized in foci upon coexpression (95% cells; Fig. 4A and fig. S6), in keeping with our previous findings (*11*). SYCE1pof formed similar or slightly reduced numbers of foci that equally colocalized with SIX6OS1, indicating a retention of SIX6OS1 binding (89% cells; Fig. 4A). We further demonstrated a similar coimmunoprecipitation of SIX6OS1 by WT SYCE1 and SYCE1pof upon coexpression in human embryonic kidney (HEK) 293 cells (Fig. 4B). Thus, the SYCE1-SIX6OS1 interaction is retained in the SYCE1 POF mutation. Could other disrupted functions contribute to the effect of the POF mutation? The only other known SYCE1 interactor is SYCE3, which undergoes low-affinity binding, as determined by its dissociation during purification (fig. S7, A and B). In contrast with the WT protein, the expression of SYCE1pof (cytoplasmic foci) in COS7 cells failed to recruit SYCE3 (preferentially nuclear) to their cytoplasmic foci (colocalization between SYCE3 and SYCE1 was observed for 95% of cells expressing WT SYCE1 and 21% of cells expressing SYCE1pof; Fig. 4C and fig. S6). Similarly, SYCE1pof failed to coimmunoprecipitate SYCE3 upon coexpression in HEK293 cells (Fig. 4D). Thus, while the SYCE1-SIX6OS1 complex is retained, the low-affinity SYCE1-SYCE3 complex is largely abolished in the SYCE1 POF mutation.

### SYCE1core undergoes conformational change to form a 1:1 complex with SIX6OS1

**Fig. 5:**
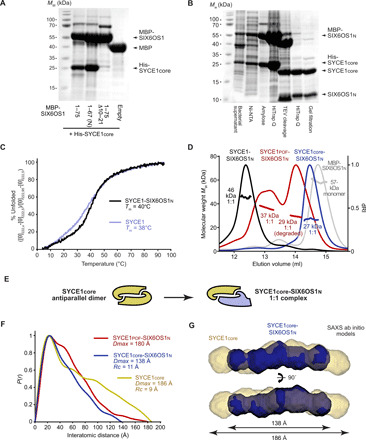
SYCE1core undergoes conformational change to form a 1:1 complex with SIX6OS1n. (**A**) Amylose pulldown following coexpression of MBP-SIX6OS1 1–75, 1–67, 1–75 Δ10–21, and free MBP with His-SYCE1core. (**B**) SDS-PAGE of the copurification of the SYCE1core-SIX6OS1n complex. Ni-NTA, Ni–nitrilotriacetic acid. (**C**) CD thermal denaturation recording the CD helical signature at 222 nm between 5° and 95°C, as % unfolded; estimated melting temperatures (*T*_m_) are indicated. (**D**) SEC-MALS analysis. SYCE1core-SIX6OS1n (blue), SYCE1_POF_-SIX6OS1n (red) and full-length SYCE1-SIX6OS1n (black) are 1:1 complexes of 27, 37 (29 kDa for the degradation product complex), and 46 kDa, respectively (theoretical 1:1 to 27, 36, and 48 kDa), while MBP-SIX6OS1n (gray) is a 57-kDa monomer (theoretical, 53 kDa). SDS-PAGE of the SYCE1_POF_-SIX6OS1n sample is shown in Fig. 1C. (**E**) Schematic of the conformational change of the SYCE1core antiparallel dimer (yellow) into a 1:1 SYCE1core-SIX6OS1n complex (yellow-blue). (**F** and **G**) SEC-SAXS analysis. (F) SEC-SAXS *P*(*r*) interatomic distance distributions of SYCE1core-SIX6OS1n (blue), SYCE1_POF_-SIX6OS1n (red), and SYCE1core (yellow), revealing maximum dimensions (*Dmax*) of 138, 180, and 186 Å, respectively. Their cross-sectional radii (*Rc*) are indicated (fig. S7D). (G) SAXS ab initio models of SYCE1core-SIX6OS1n (blue) and SYCE1core (yellow); averaged models were generated from 20 independent DAMMIF runs. Data for SYCE1core and full-length SYCE1 are reproduced from (*28*).

What is the molecular basis of SIX6OS1 binding by SYCE1? As this is retained in SYCE1pof, we reasoned that SIX6OS1 binding must be mediated by SYCE1’s structural core. We screened SYCE1core against a library of SIX6OS1 constructs through bacterial coexpression and identified a robust interaction with amino acids 1 to 67 of SIX6OS1, herein referred to as SIX6OS1_N_ (Figs. 1B and 5A). We were able to purify the SYCE1core-SIX6OS1_N_ complex by reciprocal affinity chromatography, ion exchange, and size exclusion chromatography (Fig. 5B) and found it to be stable under all experimental conditions tested. We were further able to purify similar complexes for SYCE1pof (with the same degradation product as upon isolated expression) and full-length SYCE1 (Fig. 1C and fig. S1B), confirming that SIX6OS1 binding is retained by all constructs containing the 25–179 core. CD analysis revealed similar α-helical content for SYCE1-SIX6OS1_N_ complexes as for their isolated SYCE1 proteins (fig. S1C). CD thermal denaturation revealed slightly increased cooperativity of unfolding and melting temperatures for SYCE1-SIX6OS1_N_ complexes relative to their isolated SYCE1 proteins (increasing from 39° to 43°C, 39° to 41°C, and 38° to 40°C for SYCE1core, SYCE1pof, and full length, respectively; Fig. 5C and fig. S1D). SEC-MALS analysis revealed that all three SYCE1-SIX6OS1_N_ complexes are 1:1, with molecular weights of 27, 37, and 46 kDa, respectively (Fig. 5D and fig. S7C). Thus, the SYCE1core undergoes conformation change from an antiparallel homodimer to a 1:1 complex upon binding to SIX6OS1_N_ (Fig. 5E).

We analyzed the conformation of the SYCE1core-SIX6OS1_N_ complex by size exclusion chromatography small-angle x-ray scattering (SEC-SAXS; fig. S7, D and E). The SAXS real-space pair-distance *P*(*r*) distribution (the distribution of interatomic distances within a protein structure) demonstrates positive skew, indicating that SYCE1core-SIX6OS1_N_ retains the rod-like structure of SYCE1core, but with a reduction in its molecular length from 186 to 138 Å (Fig. 5F). Furthermore, its cross-sectional radius is slightly increased from 9 to 11 Å (fig. S7F), suggesting an increase from a two- to four-helical coiled coil. These geometric changes are consistent with the SYCE1core-SIX6OS1_N_ 1:1 complex forming a shorter but wider coiled coil than the isolated SYCE1core dimer, as indicated by their SAXS ab initio models (Fig. 5G). Furthermore, the SAXS *P*(*r*) distribution of SYCE1pof indicates a similar elongated structure but with an increased tail to a maximum dimension of 180 Å (Fig. 5F), consistent with it containing the same SYCE1core-SIX6OS1_N_ structure with an extended and potentially unstructured C terminus to amino acid 240. We conclude that SYCE1core mediates a direct interaction with SIX6OS1_N_ that imposes a conformational change to a 1:1 complex that adopts a shorter and wider coiled-coil conformation than the isolated SYCE1core antiparallel homodimer.

### SYCE1_POF_ disrupts a second SYCE1-SIX6OS1 binding interface

**Fig. 6:**
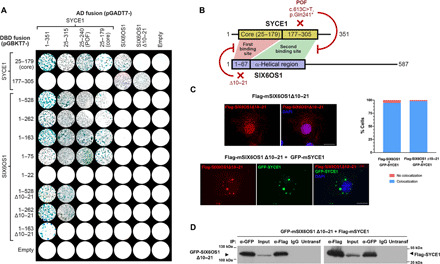
SYCE1 undergoes multivalent interaction with SIX6OS1 in yeast, but SIX6OS1 Δ10–21 retains SYCE1 binding in heterologous systems. (**A**) Y2H analysis of interactions between SYCE1 and SIX6OS1 in which positive reactions are indicated by the growth of blue colonies. These data are representative of three repeats. (**B**) Schematic of the SYCE1-SIX6OS1 interaction based on the Y2H data in (A), with the two binding sites highlighted in red and green. The SYCE1 POF mutation blocks the second binding interface between SYCE1 177–305 and SIX6OS1 downstream sequence within region 1–262, whereas the SIX6OS1 Δ10–21 deletion blocks the first binding interface between SYCE1core (25–179) and SIX6OS1n (1–67). (**C**) COS7 cells were transfected with mouse *Six6os1* Δ*10–21* alone or in combination with mouse *Syce1*. SIX6OS1 Δ10–21 showed nuclear localization with some cytoplasmatic signal and colocalized in cytoplasmic foci with SYCE1; the percentage of cells exhibiting colocalization is shown. Scale bars, 20 μm. (**D**) Coimmunoprecipitation of SIX6OS1 Δ10–21 and Flag-SYCE1 from cotransfected HEK293T cells using anti-Myc or anti-EGFP antibodies, or mouse IgG as a negative control. SIX6OS1 Δ10–21 coimmunoprecipitated SYCE1, indicating that the second SYCE1 binding interface is retained. The untransfected lanes confirm the absence of SIX6OS1 Δ10–21 and SYCE1 in total protein extracts of untransfected 293T cells.

Does the SYCE1core-SIX6OS1_N_ complex represent the sole means by which SYCE1 interacts with SIX6OS1? We were unable to obtain soluble biochemical complexes containing SIX6OS1 sequences beyond its N terminus and so used yeast two-hybrid (Y2H) to test SYCE1 binding by full-length SIX6OS1. Having confirmed direct binding of SYCE1core to full-length SIX6OS1, we used C-terminal truncation to dissect its minimal binding site to amino acids 1 to 75, in keeping with our biochemical findings, and identified an additional interaction between SYCE1 177–305 and full-length SIX6OS1 (Fig. 6A).

To establish whether SYCE1core and 177–305 bind to the same or distinct sites within SIX6OS1, we established an internal deletion of SIX6OS1 amino acids 10 to 21 (Δ10–21) that blocks formation of the SYCE1core-SIX6OS1_N_ biochemical complex (Fig. 5A). SIX6OS1 1–22 did not interact with any SYCE1 construct (Fig. 6A), indicating that amino acids 10 to 21 are necessary but not sufficient for SYCE1core binding. While Δ10–21 completely abrogated the Y2H interaction of full-length SIX6OS1 with SYCE1core (25–179), it retained a robust interaction with SYCE1 177–305, suggesting distinct SIX6OS1-binding sites (Fig. 6A). Furthermore, Δ10–21 blocked the ability of SIX6OS1 1–262 to interact with SYCE1core and SYCE1pof (amino acids 25 to 240) while retaining its binding to full-length and 25–315 SYCE1 (Fig. 6A). Thus, SYCE1 undergoes multivalent interactions with SIX6OS1, with the first binding interface mediated by SYCE1core and SIX6OS1_N_ (1–67), and the second interface mediated by SYCE1 177–305 and downstream sequence within SIX6OS1 1–262. Furthermore, the first and second binding interfaces are specifically disrupted by SIX6OS1 deletion Δ10–21 and the SYCE1 POF mutation, respectively, and in both cases, an SYCE1-SIX6OS1 complex is retained through the unaffected alternative site (Fig. 6B).

### SIX6OS1 Δ10–21 retains SYCE1 binding in heterologous systems

Our biochemical and Y2H analyses concluded that SIX6OS1 Δ10–21 would disrupt the first SYCE1-SIX6OS1 binding interface while retaining complex formation through the second interface. In support of this, we found that SIX6OS1 Δ10–21 retained its ability to form intense colocalized foci with SYCE1 upon coexpression in COS7 cells (98% of the cells; Fig. 6C), similar to our previous observations for the SYCE1 POF mutation (Fig. 4A). Similarly, SIX6OS1 Δ10–21 retained its ability to coimmunoprecipitate SYCE1 upon coexpression in HEK293 cells (Fig. 6D). Thus, localization and coimmunoprecipitation data from heterologous systems support our Y2H findings that the second SYCE1-SIX6OS1 binding interface is retained in SIX6OS1 Δ10–21, mirroring the retention of only the second binding interface that is predicted for the 126–155 deletion of the SYCE1 c.375-2A>G NOA mutation (*40*).

### SIX6OS1 Δ10–21 leads to failure of SC assembly and murine infertility

**Fig. 7:**
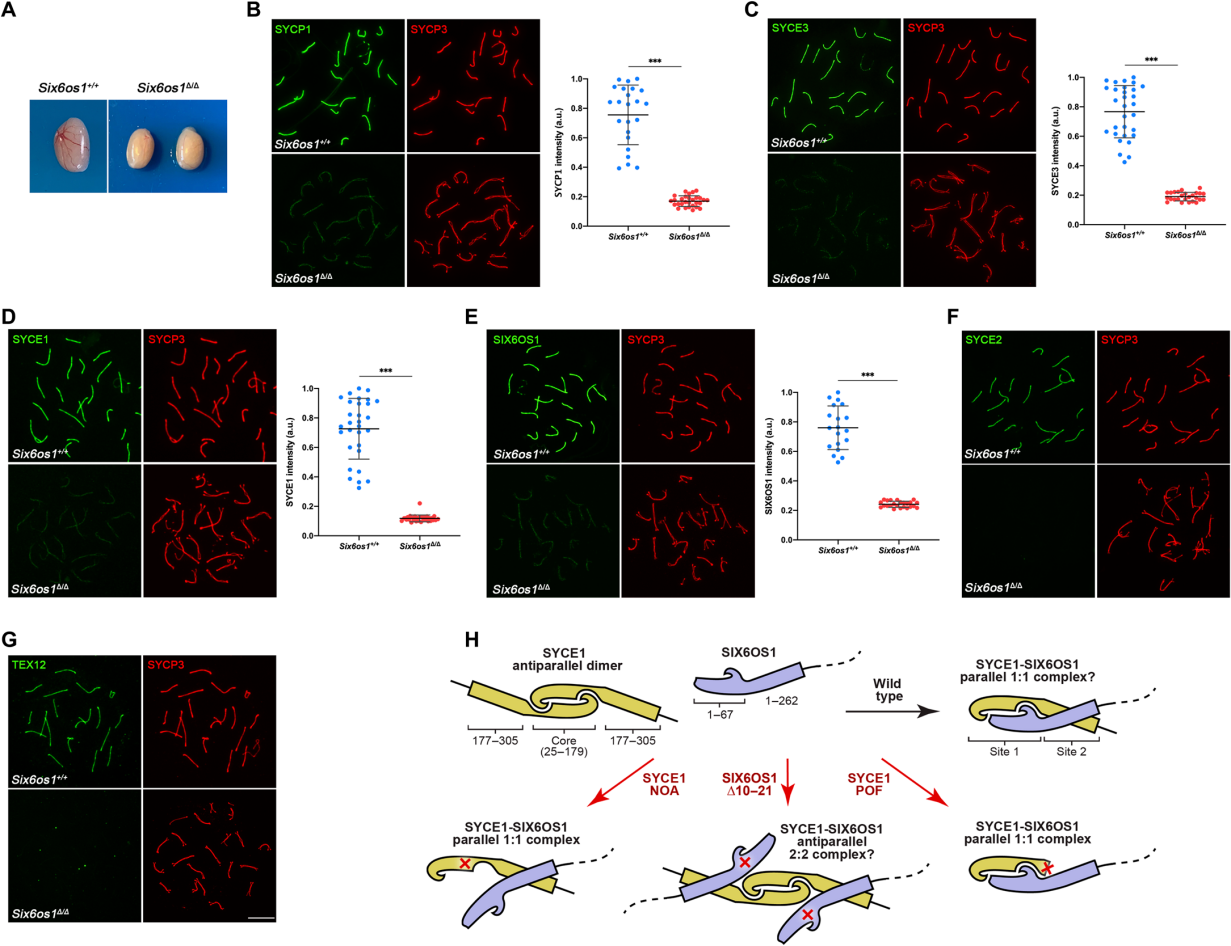
Synapsis between homologs is disrupted in *Six6os1*^Δ*10–21/*Δ*10–21*^ spermatocytes. (**A**) Genetic deletion of amino acids 10 to 21 of SIX6OS1 led to a reduction of the testis size compared to the WT (mice of 3 months of age). (**B**) Double immunolabeling of WT pachytene and *Six6os1*^Δ*/*Δ^ zygotene-like spermatocytes with SYCP3 (red) and SYCP1 (green). AEs failed to synapse in *Six6os1*^Δ*/*Δ^ spermatocytes despite partial alignment, with reduced loading of SYCP1 along the AEs. (**C** to **G**) Double immunolabeling of spermatocyte spreads with SYCP3 (red) and all CE components (green). *Six6os1*^Δ*/*Δ^ zygotene-like spermatocytes showed reduced signals of (C) SYCE3, (D) SYCE1, and (E) SIX6OS1, and the absence of (F) SYCE2 and (G) TEX12 from the AEs. Scale bars, 10 μm. Plots represent the quantification of fluorescence intensity levels in *Six6os1*^Δ*/*Δ^ zygotene-like and WT pachytene spermatocytes (B to E). Welch’s *t* test analysis: ***P < 0.0001. (**H**) Schematic of how the SYCE1 antiparallel dimer (yellow) undergoes conformational change upon interaction with SIX6OS1 (blue) to form a possible 1:1 complex through consecutive binding interfaces mediated by SYCE1core-SIX6OS1n (site 1) and SYCE1 177–305 and downstream sequence within SIX6OS1 1–262 (site 2). The consequence of SYCE1 mutations associated with POF (c.613C>T) and NOA (c.375-2A>G) and SIX6OS1 Δ10–21 on the integrity, predicted stoichiometry, and conformation of resultant SYCE1-SIX6OS1 complexes is illustrated. Photo credit (A): Laura Gómez-H, Instituto de Biología Celular y Molecular del Cáncer.

Having established that the severe phenotype of the SYCE1 POF mutation likely results from the disruption of the second SYCE1-SIX6OS1 binding interface and its interaction with SYCE3, we wondered whether a similar phenotype would result from the sole disruption of the first SYCE1-SIX6OS1 binding interface. To test this, we generated mice harboring mutations of *Six6os1* alleles encoding internal in-frame deletions of amino acids 10 to 21 (equivalent numbering to the human protein) (fig. S8, A and B). While heterozygotes (designated *Six6os1*^Δ*10–21/WT*^) were fertile, both male and female homozygotes (designated *Six6os1*^Δ*10–21/*Δ*10–21*^) were infertile, similar to the SYCE1 POF mutation. In males, we observed reduced testis size (Fig. 7A) and a zygotene-like arrest similar to that observed in the *Six6os1* and *Syce1* knockouts (*11*, *16*). The mutant spermatocytes were defective in synapsis and SC assembly, with reduced staining for SC proteins SYCP1 (Fig. 7B) and SYCE3 (Fig. 7C) and no staining for SYCE2-TEX12 (Fig. 7, F and G). In contrast with their complete absence in the SYCE1 POF mutation, we observed some residual staining for SYCE1 (Fig. 7D) and SIX6OS1 (Fig. 7E) even though the levels of transcription of *Six6os1*^Δ*10–21*^ appeared to be increased in the mutant testis (fig. S9 and table S1B). We detected γ-H2AX (fig. S10A) and DMC1/RAD51 foci (fig. S10, B and C) on aligned axial elements but no MLH1 foci (fig. S10D), indicating the proper induction of DSBs with their failed repair and absence of COs. Thus, SIX6OS1 Δ10–21 leads to infertility with a phenotype of failed DSB repair and SC assembly, similar to the SYCE1 POF mutation and those reported for disruption of structural components of the CE (*10*, *11*, *16*–*18*).

Thus, we conclude that both first and second SYCE1-SIX6OS1 binding interfaces are essential for SC assembly and meiotic progression. Furthermore, these findings explain how the sole disruption of individual SYCE1-SIX6OS1 binding interfaces by SYCE1 NOA (c.375-2A>G) and POF (c.613C>T) mutations result in the reported familial cases of human infertility.

## DISCUSSION

The structural and functional integrity of the SC is contingent on the structure and assembly of is constituent protein components. Here, we report that SC assembly depends on multivalent interactions between CE components SYCE1 and SIX6OS1 that are disrupted by infertility-associated mutations of SYCE1. The first binding interface is formed by the structural core of SYCE1 (SYCE1core; amino acids 25 to 179), which undergoes conformational change from an antiparallel homodimer to a 1:1 complex upon interaction with SIX6OS1’s N terminus (SIX6OS1_N_; amino acids 1 to 67). The second binding interface is formed by downstream sequence within SIX6OS1 1–262 interacting directly with SYCE1 177–305. Through the generation of mice harboring an internal deletion of SIX6OS1’s N terminus (Δ10–21) and the SYCE1 POF mutation (murine p.Gln243*), which specifically block the first and second binding interfaces, respectively, we find that integrity of both SYCE1-SIX6OS1 binding interfaces is essential for SC assembly and meiotic progression in vivo.

What is the structure of the SYCE1-SIX6OS1 complex? SEC-SAXS analysis revealed that the SYCE1core-SIX6OS1_N_ 1:1 complex formed by the first binding interface has a length and cross-sectional radius of 138 and 11 Å, in comparison with 186 and 9 Å for the SYCE1core dimer. We previously reported a model for SYCE1core in which amino acids 52 to 179 form an antiparallel dimeric coiled coil containing a midline “kink”, with α helices of amino acids 25 to 50 packing against this structural core (fig. S11A) (*28*). A maximum dimension of 138 Å for SYCE1core-SIX6OS1_N_ suggests a coiled-coil length of approximately 92 amino acids, given a helical rise of 1.5 Å per amino acid (*44*). This could be explained by the 52–179 region forming a helix-turn-helix structure through exaggeration of the kink to a full turn, which may combine with the α helix formed by amino acids 25 to 50 and an α helix from SIX6OS1_N_ to form a four-helical coiled coil, consistent with its 11-Å cross-sectional radius (fig. S11B). The second binding interface between SYCE1 177–305 and downstream sequence within SIX6OS1 1–262 suggests that SYCE1core-SIX6OS1_N_ likely adopts a parallel configuration to form a single SYCE1-SIX6OS1 1:1 complex of consecutive first and second binding interfaces (Fig. 7H).

Our analysis of the SYCE1-SIX6OS1 complex reveals how the three reported clinical mutations of SYCE1 differentially affect its interaction with SIX6OS1. The SYCE1 NOA mutation c.197-2A>G is predicted to result in a truncated product of amino acids 1 to 65 (*39*), which would disrupt both binding sites and so likely abrogates SYCE1-SIX6OS1 complex formation and thus works as a null mutation. The SYCE1 NOA mutation c.375-2A>G is predicted to result in internal deletion of amino acids 126 to 155 (*40*), which would disrupt the first binding interface while retaining the second binding interface, and so is likely to result in a conformationally altered 1:1 complex (Fig. 7H). In contrast, while Δ10–21 SIX6OS1 similarly disrupts the first binding interface and retains the second binding interface, the SYCE1core remains unaffected and so is predicted to enable formation of a head-to-head 2:2 complex (Fig. 7H). The SYCE1 POF mutation c.613C>T generates a premature stop codon (p.Gln241*) that gives a truncated product of amino acids 1 to 240 (*41*), which we have demonstrated disrupts the second binding interface while retaining the first binding interface (Fig. 7H). Thus, the latter two infertility-associated mutations of SYCE1 specifically disrupt one SYCE1-SIX6OS1 interface while retaining the other, which combine with our mouse genetic studies to confirm that both interfaces are essential for the structural assembly of the SC and its function in meiosis.

What are the structural roles of SYCE1 and SYCE1-SIX6OS1 within the SC? Our analyses of *Syce1^POF/POF^* and *Six6os1*^Δ*10–21/* Δ*10–21*^ mouse strains revealed similar phenotypes with retention of some SYCP1 and SYCE3 recruitment to chromosome axes, with absence or substantial reduction of SYCE1 and SIX6OS1, and lack of recruitment of SYCE2-TEX12. This pattern suggests a hierarchical model of SC assembly in which SYCE1 and SYCE1-SIX6OS1 lie downstream of SYCP1 and SYCE3, and upstream of SYCE2-TEX12 (Fig. 1A), which is consistent with existing knockout data (*10*, *11*, *15*–*18*). The disruption of SYCE3 binding by the POF mutation suggests that its SYCE1-SIX6OS1 complex would be defective for SC recruitment, whereas the SYCE1-SYCE3 interaction, and hence SC recruitment, should be retained for the SYCE1-SIX6OS1 complex of the SIX6OS1 Δ10–21 internal deletion. This explains the greater severity of the CE loading defect in *Syce1^POF/POF^* than *Six6os1*^Δ*10–21/* Δ*10–21*^, in which SYCE1 and SIX6OS1 staining was substantially reduced in the latter (83.77% of SYCE1 reduction, 0.12 ± 0.02 in the *Six6os1*^Δ*10–21/* Δ*10–21*^ versus 0.73 ± 0.21 in the WT; 68.27% of SIX6OS1 reduction, 0.24 ± 0.02 in the *Six6os1*^Δ*10–21/* Δ*10–21*^ versus 0.76 ± 0.15 in the WT) but completely absent in the former. Thus, we conclude that the first and second SYCE1-SIX6OS1 interfaces are essential for initiation of SC CE formation and likely function by stabilizing a local three-dimensional SC structure that mediates recruitment and self-assembly of SYCE2-TEX12 into fibers that mediate SC elongation along the chromosome axis. Furthermore, the SYCE1 POF mutation is likely worsened by its additional disruption of SYCE3 binding that removes the residual SYCE1-SIX6OS1 SC recruitment observed for the SIX6OS1 Δ10–21 internal deletion.

The existence of SYCE1core as an isolated antiparallel homodimer and in a 1:1 complex with SIX6OS1_N_ raises the question of which is the biologically relevant conformation. It is important to highlight that the CD melting temperatures of SYCE1-SIX6OS1_N_ complexes and isolated SYCE1 dimers are very similar, ranging between 38° and 41°C. In contrast, highly stable SC components SYCE2-TEX12 and SYCP3 have melting temperatures of approximately 65°C (*24*, *29*). Thus, the relatively low melting temperatures of SYCE1-SIX6OS1_N_ complexes and SYCE1 suggest that they may undergo conformational change in vivo, with each conformation functioning at different stages of meiosis and/or at different locations within the SC. Furthermore, our analysis of SYCE1 infertility-associated mutations and a targeted internal deletion of SIX6OS1 revealed at least four possible conformations of SYCE1 and SYCE1-SIX6OS1 complexes (Fig. 7H). Owing to the direct competition between SIX6OS1_N_ binding and SYCE1core dimerization, these conformations could be achieved in the absence of mutations, through alterations of protein levels, local concentrations, allosteric changes, and posttranslational modifications. Hence, alterative conformations of SYCE1 and SYCE1-SIX6OS1 are intriguing candidates for local structural heterogeneity and the propagation of signals along the length of the SC, which could function in roles such as crossover enforcement and interference. Thus, as we progress toward a full molecular understanding of the mammalian SC, the multivalent SYCE1-SIX6OS1 interactions described herein provide tantalizing possibilities for a dynamic role of SC structure in its enigmatic functions in the mechanics of meiosis.

## MATERIALS AND METHODS

### Recombinant protein expression and purification

Human SYCE1 sequences were cloned into pHAT4 and pMAT11 vectors (*45*) for bacterial expression as His- and His-MBP (Maltose-Binding Protein) fusions with TEV (Tobacco Etch Virus) cleavage sites for fusion protein removal. Human SIX6OS1 was cloned into pRSF-Duet1 vectors with a TEV-cleavable N-terminal MBP fusion for coexpression with SYCE1. Proteins were expressed in BL21(DE3) *Escherichia coli* cells (Novagen), in 2xYT (Yeast Extract Tryptone) media. Expression was induced with addition of 0.5 mM isopropyl-β-d-thiogalactopyranoside with the cells incubated at 25°C for 16 hours. Cells were lysed via sonication in 20 mM tris (pH 8.0) and 500 mM KCl, followed by centrifugation. Supernatant was applied to an amylose (New England Biolabs) affinity chromatography column, followed by HiTrap Q HP (GE Healthcare) anion exchange chromatography. His- and His-MBP/MBP tags were removed by incubation with TEV protease at 4°C for 16 hours. The cleaved proteins were further purified by HiTrap Q HP (GE Healthcare) anion exchange chromatography followed by size exclusion chromatography (HiLoad 16/600 Superdex 200, GE Healthcare). The purified proteins/complexes were concentrated using Microsep Advance 3 kDa (PALL) centrifugal filter units and stored at −80°C. Protein samples were analyzed for purity using Coomassie-stained SDS–polyacrylamide gel electrophoresis. Protein molecular weights and extinction coefficients were calculated using ExPASY ProtParam (http://web.expasy.org/protparam/) with protein concentrations determined using a Cary 60 ultraviolet (UV) spectrophotometer (Agilent).

### Circular dichroism

Far-UV CD spectra were collected using a Jasco J-810 spectropolarimeter (Institute for Cell and Molecular Biosciences, Newcastle University). Wavelength scans were recorded at 4°C from 260 to 185 nm at 0.2-nm intervals using a 0.2-mm path length quartz cuvette (Hellma). Protein samples were measured at 0.2 to 0.4 mg/ml in 10 mM Na_2_HPO_4_ (pH 7.5) and 150 mM NaF. Nine measurements were taken for each sample, averaged, buffer-corrected and converted to mean residue ellipticity (MRE) ([θ]) (×1000 deg·cm^2^·dmol^−1^ per residue). Spectral deconvolutions were carried out using the Dichroweb CDSSTR algorithm (http://dichroweb.cryst.bbk.ac.uk). CD thermal melts were recorded at 222 nm between 5° and 95°C, at intervals of 0.5°C with a 1°C/min ramping rate. Protein samples were measured at 0.1 mg/ml in 20 mM tris (pH 8.0), 150 mM KCl, and 2 mM dithiothreitol (DTT), using a 1-mm path length quartz cuvette (Hellma). The data were plotted as % unfolded after conversion to MRE ([θ]_222,*x*_-[θ]_222,5_)/([θ]_222,95_-[θ]_222,5_). The melting temperature was determined as the temperature at which the proteins are 50% unfolded.

### Size exclusion chromatography multiangle light scattering

SEC-MALS analysis of protein samples was carried out at concentrations of 5 to 20 mg/ml in 20 mM tris (pH 8.0), 150 mM KCl, and 2 mM DTT. Samples were loaded onto a Superdex 200 Increase 10/300 GL (GE Healthcare) column at 0.5 ml/min using an ÄKTA Pure (GE Healthcare) system. The eluate was fed into a DAWN HELEOS II MALS detector (Wyatt Technology), followed by an Optilab T-rEX differential refractometer (Wyatt Technology). SEC-MALS data were collected and analyzed using ASTRA 6 software (Wyatt Technology), using Zimm plot extrapolation with a 0.185 ml/g dn/dc value to determine absolute protein molecular weights.

### Size exclusion chromatography small-angle x-ray scattering

SEC-SAXS experiments were carried out on beamline B21 at the Diamond Light Source synchrotron facility (Oxfordshire, UK). Protein samples at concentrations 6 to 20 mg/ml were loaded onto a Superdex 200 Increase 10/300 GL size exclusion chromatography column (GE Healthcare) in 20 mM tris (pH 8.0) and 150 mM KCl at 0.5 ml/min using an Agilent 1200 high-performance liquid chromatography system. The eluate was fed through the experimental cell, with SAXS data recorded at 12.4 keV, in 3.0-s frames with a detector distance of 4.014 m. ScÅtter 3.0 (www.bioisis.net) was used to subtract and average the frames and carry out the Guinier analysis for the *Rg* and cross-sectional *Rg* (*Rc*). *P*(*r*) distributions were fitted using PRIMUS. Ab initio modeling was performed using DAMMIF (*46*) imposing P1 symmetry. Twenty independent runs were averaged. The PyMOL Molecular Graphics System, Version 2.0 Schrödinger, LLC was used to generate images of the SAXS ab initio models.

### Yeast two-hybrid

Constructs of human SYCE1 and SIX6OS1 were cloned into pGBKT7 and pGADT7 vectors (Clontech). Y2H experiments were carried out using the Matchmaker Gold system (Clontech) according to the manufacturer’s guidelines. Y187 yeast strain was transformed with pGBKT7 vectors, while the Y2H gold strain was transformed with pGADT7 vectors. Yeast transformations were carried out using standard lithium acetate methods. Mating of the two strains was carried out in 0.5 ml 2× YPDA (Yeast Peptone Dextrose Adenine) at 30°C, 40 rpm, by mixing respective colonies. After 24 hours, the cultures were centrifuged and pellets were resuspended in 0.5xYPDA. These were then plated onto SD/−Trp/−Leu to select for mated colonies and onto SD/−Trp/−Leu/–Ade/−His with X-α-gal to detect mated colonies through ADE1, HIS3, and MEL1 reporter gene activation. Plates were then incubated for 5 days at 30°C.

### Production of CRISPR-Cas9–edited mice

For developing the *Syce1^POF/POF^* model, *Syce1*–single-guide RNA (sgRNA) 5′-TGACTTCTTTCCACACTATC-3′ targeting the intron 10 was predicted at https://eu.idtdna.com/site/order/designtool/index/CRISPR_SEQUENCE. This crRNA (CRISPR RNA), the tracrRNA (trans-activating CRISPR RNA), and the ssODN (single-stranded donor oligonucleotides) (5′-GGGACTCTTCCTCCGAAGCCATGAGGCAGCTGCAGCAATGTAAGATGCAGGGTGGGGCAGGAGGAGGAAATGTCTAGCACTGACTTCTTTCCACACCCCCAGGTAGATCTTCAAGGATGAGAACAAGAAAGCTGAGG

AGTTCCTAGAGGCTGCAGCTCAGCAGCACGAGCAGCTGCAGCAGAGGTGCCACCAGCTACAG-3′) were produced by chemical synthesis at IDT. The ssODN contains the mutated base (C>T, p.Gln241*) and the peptidyl-glycine α-amidating monooxygenase (PAM) was mutated by substituting it by the human intron sequence (ACTATCAG > CCCCCAG). The crRNA and tracrRNA were annealed to obtain the mature sgRNA. A mixture containing the sgRNAs, recombinant Cas9 protein (IDT), and the ssODN [Cas9 (30 ng/μl), annealed sgRNA (20 ng/μl each), and ssODN (10 ng/μl)] were microinjected into B6/CBA F2 zygotes (hybrids between strains C57BL/6 J and CBA/J) (*47*) at the Transgenic Facility of the University of Salamanca. Edited founders were identified by polymerase chain reaction (PCR) amplification (Taq polymerase, NZYTech) with primers flanking the exon 11 (primer F 5′-CTGTAGAGAAACTGATGAAAGT-3′ and R 5′-CAAGAAAATATGAAGAGACATAC-3′) producing an amplicon of 398 base pairs (bp) for both edited and WT alleles, and either direct sequenced or subcloned into pBlueScript (Stratagene) followed by Sanger sequencing, selecting the point mutation in the targeted region of *Syce1* (fig. S2). For generating the *Six6os1*^Δ*10–21/* Δ*10–21*^ (named as *Six6os1*^Δ*/*Δ^), *Six6os1*-crRNA G68 5′-ATCTGTTTGTCAGTTTGGAC-3′ and *Six6os1*-crRNA G75 5′-TACTTATGTCTTGCTCATAC-3′ targeting exons 2 and 3 and the ssODN (5′-GTTCTTACTTTATGTATGCTCTTTTATATATGGCTTCTGAAAGTTTTATTATTTATTTTACACAGTGTCCAAGATGAATGATAATCTGTTTGTCAGTTTGCAAGACATAAGTATTAAAGAAGATACGATTCAAAGAATTAATAGTAAGTAGTTTTGCATGAAATAAATATTTTAGTCTTTTGGTTTTATCTTATATAGCA-3′) were predicted, produced, and microinjected, as previously described. Edited founders with the predicted deletion were identified through PCR using primers flanking this region (primer F 5′-CACTTACATTTTCCTTTTAAGAATGC-3′ and R 5′-CCCCTCTCATACATACAAGTTGC-3′). The Δ10–21 allele was 285 bp long versus 413 bp of the WT allele (fig. S8, A and B). The founders were crossed with WT C57BL/6 J to eliminate possible unwanted off-targets. Heterozygous mice were resequenced and crossed to give rise to edited homozygous. Genotyping was performed by analysis of the PCR products of genomic DNA with primers F and R.

#### Histology

For histological analysis of ovaries, after the necropsy of the mice, their ovaries were removed and fixed in formol 10%. They were processed into serial paraffin sections and stained with hematoxylin and eosin. The samples were analyzed using a microscope OLYMPUS BX51, and images were taken with a digital camera OLYMPUS DP70.

#### Immunocytology

Testes were detunicated and processed for spreading using a conventional “dry-down” technique. Oocytes from fetal ovaries (E17.5 embryos) were digested with collagenase, incubated in hypotonic buffer, disaggregated, and fixed in paraformaldehyde. Both meiocyte preparations were incubated with the following primary antibodies for immunofluorescence (IF): rabbit αSIX6OS1 R1 and R2 [1:100, Proteogenix (*11*)], rabbit αSYCE1 17406-1-AP (1:50, Proteintech), guinea pig αSYCE1 (1:100, provided by C. Höög), mouse αSYCP3 immunoglobulin G (IgG) sc-74569 (1:1000, Santa Cruz Biotechnology), rabbit serum αSYCP3 K921 (1:500), rabbit αSYCP1 IgG ab15090 (1:200), guinea pig αSYCE3(1:20, provided by R. Benavente), guinea pig αSYCE2 (1:100, provided by C. Höög), rabbit αTEX12 IgG (1:100, provided by R. Benavente), rabbit anti-γ-H2AX (ser139) IgG #07-164 (1:200) (Millipore), mouse αMLH1 51-1327GR (1:5, BD Biosciences), rabbit αRAD51 PC130 (1:50, Calbiochem), and rabbit αDMC1 R1 and R2 (1:500, Proteogenix). The secondary antibodies used were goat Alexa 555 α-mouse A-32727, goat Alexa 488 α-mouse A-11001, donkey Alexa 555 α-rabbit A-31572 (1:200, Thermo Fisher Scientific), goat Alexa 488–Fab α-rabbit 111-547-003, and donkey fluorescein isothiocyanate α–guinea pig 706-095-148 (1:100, Jackson Immunoresearch). Slides were visualized at room temperature using a microscope (Axioplan 2; Carl Zeiss Inc.) with 63× objectives with an aperture of 1.4 (Carl Zeiss Inc.). Images were taken with a digital camera (ORCA-ER; Hamamatsu) and processed with OPENLAB 4.0.3 and Photoshop (Adobe). Quantification of fluorescence signals was performed using ImageJ software.

### Cell lines and transfections

HEK293T and COS7 cell lines were and obtained from the American Type Culture Collection (ATCC). Cell lines were tested for mycoplasma contamination (Mycoplasma PCR ELISA, Sigma-Aldrich). They were transfected with Jetpei (PolyPlus) according to the manufacturer’s protocol.

#### Immunoprecipitation and Western blotting

HEK293T cells were transiently transfected, and whole-cell extracts were prepared and cleared with protein G Sepharose beads (GE Healthcare) for 1 hour. The antibody was added for 2 hours, and immunocomplexes were isolated by adsorption to protein G Sepharose beads overnight. After washing, the proteins were eluted from the beads with 2× SDS gel-loading buffer 100 mM tris-HCl (pH 7), 4% SDS, 0.2% bromophenol blue, 200 mM β-mercaptoethanol, and 20% glycerol and loaded onto reducing polyacrylamide SDS gels. The proteins were detected by Western blotting with the indicated antibodies. Immunoprecipitations were performed using mouse α-Flag IgG (5 μg; F1804, Sigma-Aldrich), mouse α–green fluorescent protein (α-GFP) IgG (4 μg; CSB-MA000051M0m, Cusabio), mouse α-Myc obtained from hybridoma cell myc-1-9E10.2 ATCC (4 μg), and ChromPure mouse IgG (5 μg/1 mg protein; 015-000-003). Primary antibodies used for Western blotting were rabbit α-Flag IgG (1:2000; F7425 Sigma-Aldrich), goat α-GFP IgG (sc-5385, Santa Cruz Biotechnology) (1:3000), and rabbit α-Myc Tag IgG (1:3000; #06-549, Millipore). Secondary horseradish peroxidase–conjugated α-mouse (715-035-150, Jackson ImmunoResearch), α-rabbit (711-035-152, Jackson ImmunoResearch), or α-goat (705-035-147, Jackson ImmunoResearch) antibodies were used at 1:5000 dilution. Antibodies were detected by using Immobilon Western Chemiluminescent HRP Substrate from Millipore. Both *Syce1_POF_* and *Six6os1* Δ10–21 complementary DNAs (cDNAs) used for IF and coimmunoprecipitation experiments were reverse transcription PCR–amplified (the primers used for it were Syce1 S 5′-GAGCAGTATGGCCACCAGACC-3′ and Syce AS 5′-GAGGAGGGTATTAGGTCCTGC-3′; Six6os1 S 5′-AGTGTCCAAGATGAATGATAATCTG-3′ and Six6os1 AS 5′-GTTCAAAAATAATAACTCAAAAAAAC-3′) from total RNA extracted from *Syce1^POF/POF^* and *Six6os1*^Δ*10–21/* Δ*10–21*^ mice, respectively. PCR-amplified fragments were cloned in pcDNA3-based mammalian expression vectors with different tags (enhanced GFP or Flag) and verified by Sanger sequencing.

### Quantitative PCR

Total RNA was isolated from testis of WT and mutant mice. To analyze the expression of *Syce1* and *Six6os1* mRNAs, equal amounts of cDNA were synthesized using SuperScript II Reverse Transcriptase (Invitrogen, Life Technologies) and Oligo (dT). Quantitative PCR (qPCR) was performed using FastStart Universal SYBR Green Master Mix (ROX) (Roche) and specific forward and reverse primers: qSYCE1_F 5′-GGACATGGTGAAAAAGTTGCAG-3′ and qSYCE1_R 5′-CAGTTCCTTCTGCAGGTTGTC-3′ for *Syce1*, and qSIX6OS1_F 5′-GCTGAATGTGGAGATAAAGAG-3′ and qSIX6OS1_R 5′-AGGAGTTTCAGGAGTTTGAGG-3′ for *Six6os1*. All qPCR reactions were performed at 95°C for 10 min and then 40 cycles of 95°C for 15 s and 62°C for 1 min on the iQ5 Thermal Cycler (Bio-Rad). β-Actin was amplified as a housekeeping gene with the primers qβ-actin_F 5′-GGCACCACACCTTCTACAATG-3′and qβ-actin_R 5′-GTGGTGGTGAAGCTGTAGCC-3′.

#### Statistics

To compare counts between genotypes, we used the Welch’s *t* test (unequal variances *t* test), which was appropriate as the count data were not highly skewed (i.e., were reasonably approximated by a normal distribution) and, in most cases, showed unequal variance. We applied a two-sided test in all the cases. Asterisks denote statistical significance: *P < 0.01, **P < 0.001, and ***P < 0.0001.

## Supplementary Material

abb1660_SM.pdf

## SUPPLEMENTARY MATERIALS

Supplementary material for this article is available at http://advances.sciencemag.org/cgi/content/full/6/36/eabb1660/DC1

## Appendix

View/request a protocol for this paper from *Bio-protocol*.
