# Supplementary material for: Meiotic chromosome synapsis depends on multivalent SYCE1-SIX6OS1 interactions that are disrupted in cases of human infertility
Source: Sci Adv. 2020 Sep 2;6(36):eabb1660. doi: 10.1126/sciadv.abb1660 (PMC7467691; doi:10.1126/sciadv.abb1660)
Supplement: abb1660_SM.pdf [file abb1660_SM.pdf]

Science Advances
AAAS

advances.sciencemag.org/cgi/content/full/6/36/eabb1660/DC1

Supplementary Materials for

Meiotic chromosome synapsis depends on multivalent SYCE1-SIX6OS1 interactions that are disrupted in cases of human infertility

Fernando Sánchez-Sáez, Laura Gómez-H, Orla M. Dunne, Cristina Gallego-Páramo, Natalia Felipe-Medina, Manuel Sánchez-Martín, Elena Llano, Alberto M. Pendas*, Owen R. Davies*

*Corresponding author. Email: owen.davies@newcastle.ac.uk (O.R.D.); amp@usal.es (A.M.P.)

Published 2 September 2020, *Sci. Adv.* **6**, eabb1660 (2020)
DOI: 10.1126/sciadv.abb1660

This PDF file includes:

Figs. S1 to S11
Table S1

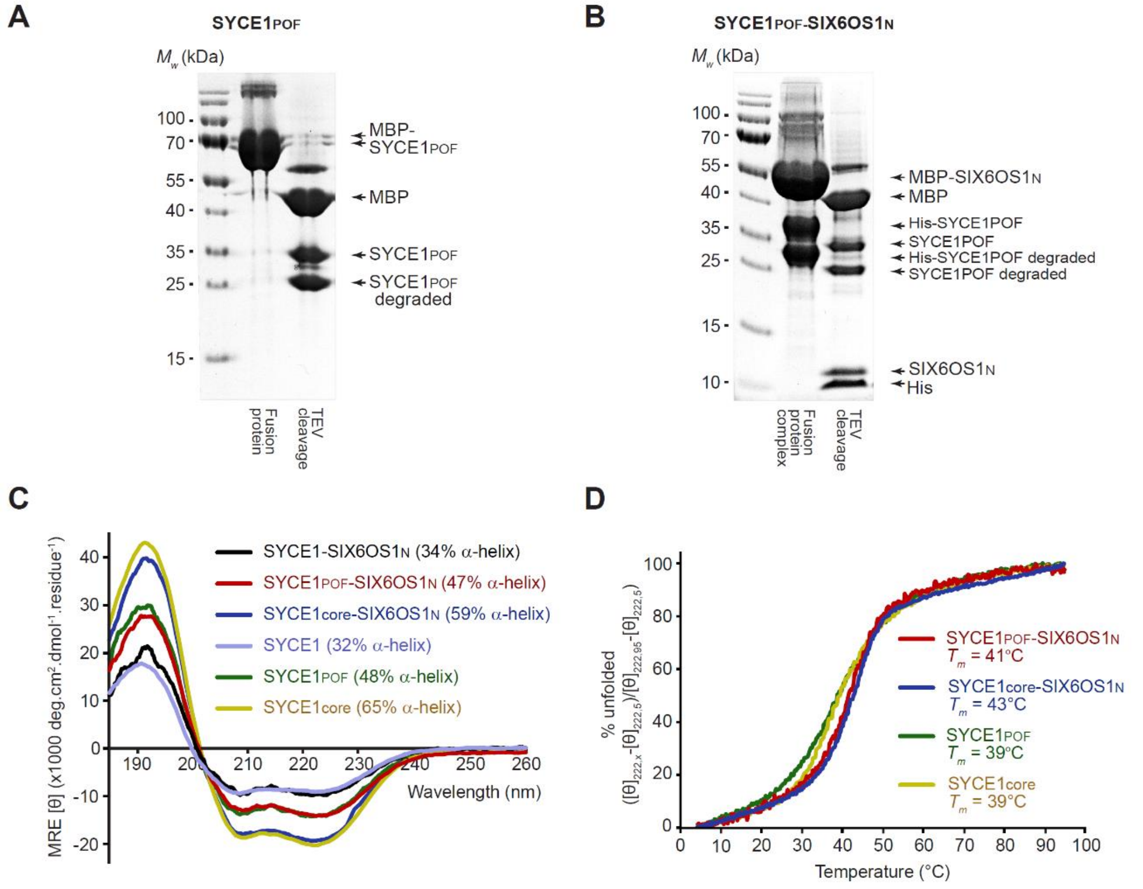

Fig. S1. Analysis of recombinant proteins and complexes.

(**A,B**) SDS-PAGE of purified fusion proteins and subsequent cleavage through incubation with TEV protease. (**A**) MBP-SYCE1POF consists of two bands of similar MW, which both undergo cleavage by TEV protease to liberate two dominant products labelled SYCE1POF and SYCE1POF degraded. (**B**) The His-SYCE1POF component of its complex with MBP-SIX6OS1N consists of two bands of equal intensity, which undergo cleavage by TEV protease to liberate two dominant products labelled SYCE1POF and SYCE1POF degraded. The TEV cleaved products of both proteins/complexes exhibit the same apparent molecular weights, which are consistent with them representing the full SYCE1POF protein and its C-terminal degradation down to the structural core of SYCE1. (**C**) Far UV circular dichroism (CD) spectra recorded between 260 nm and 185 nm in mean residue ellipticity, MRE ([θ]) (x1000 deg.cm^2.dmol^{-1}.residue^{-1}). Data were deconvoluted using the CDSSTR algorithm

revealing the helical contents indicated. (**D**) CD thermal denaturation recording the CD helical signature at 222 nm between 5°C and 95°C, as % unfolded; estimated melting temperatures (*Tm*) are indicated.

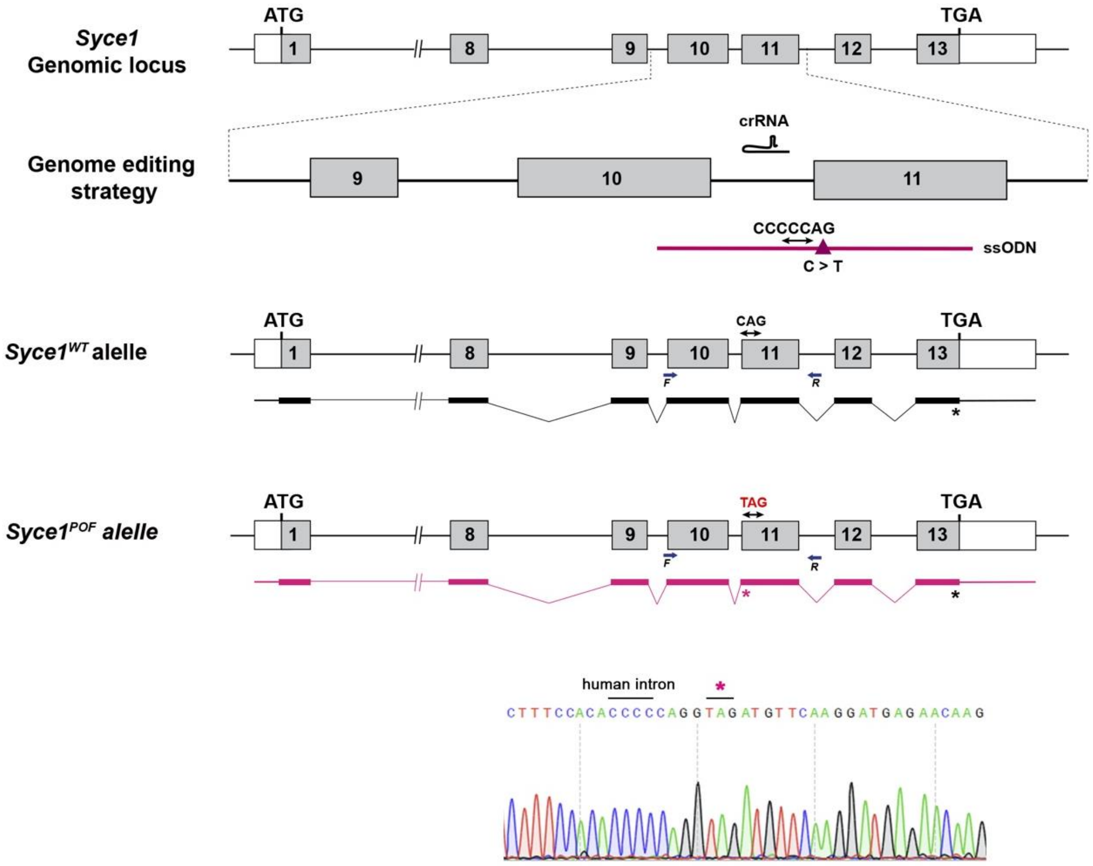

Fig. S2. Generation of *Syce1*$^{POF/POF}$ mouse model.

Diagrammatic representation of the mouse *Syce1* locus (WT) and the genome editing strategy showing the crRNA located on intron 10 and the ssODN targeting a region between exon 10 and 11 carrying the point mutation (c.613C>T, p.Gln241*) in addition to the PAM substituted by the human intron sequence (see methods, the mouse sequence caCTATcag was mutated to the human sequence caCCCCcag). The corresponding coding exons (grey boxes) and non-coding exons (open boxes) are represented. Thin (non-coding) and thick (coding sequences) lines under exons represent the expected transcript derived from WT (black) and *Syce1POF* edited allele (pink). ATG, initiation codon; TGA and *, stop codon. The newly generated stop codon in the edited transcript is indicated (*pink). Primers for PCR genotyping (F and R) are

represented by arrows. The nucleotide sequence of the edited region derived from PCR amplification of DNA from the *Syce1*$^{POF/POF}$ is indicated.

```
SP|Q8N0S2|SYCE1_HUMAN             MAGRSLTSKA--EPTAGAVDRAEKAGGQDTSSQKIEDLMEMVQKLQKVGSLEPRVEVLINR 59
SP|Q9D495|SYCE1_MOUSE             MATRPQPLGMEPEGSADLLHGPEGARGQYGSTQKIEDLMDMVKKLQKVGSLEPRIEVLINR 61
TR|A0A2I3SHS9|A0A2I3SHS9_PANTR MAGRSLTSKA--EATAGAMDRAEKAGGQDTSSQKIEDLMEMVQKLQKVGSLEPRVEVLINR 59
SP|Q32LK9|SYCE1_BOVIN             MAGRPGSS-N--AEAAGAVGPTDEARGQAESSQKIEDLMEMVKKLQKVGSLEPRVEVLINR 58
TR|F1SCT5|F1SCT5_PIG              MAGRPGPS-S--EESAGALGRAGEAGGQAKSSQKIEDLMEMVKKLQKAGSLEPKVEVLINR 58
TR|F1PG50|F1PG50_CANLF            ------------------MEPAEKAGGQAKSSQKIEDLLEMVKKLQKAGSLEPRVEVLINR 43
TR|F7BBC0|F7BBC0_ORNAN            ME-------KNQQDQRSGLGMREKAMEHTEYKVEAEDLVMLVEKLQKAGTLEPRIEDLIIK 54
TR|G3W340|G3W340_SARHA            -----------------------------------------------------KMEEMISI 8
TR|H3ALY1|H3ALY1_LATCH            FELARVPLGGKAAAVGGLLFHPSGVRGNEESRLELEDLLKIVKELKQAGKTVPRIEELVKK 61
TR|K7FPR7|K7FPR7_PELSI            ------------------------------------------------GTLEPRMDDLVGR 13
                                                                                     ::: ::

SP|Q8N0S2|SYCE1_HUMAN             INEVQQAKKKANKDLGEARTICEALQKELDSLHGEKVHLKEILSKKQETLRILRLHCQEKE 120
SP|Q9D495|SYCE1_MOUSE             INEVQQAKKKASEELGEAQTVWDNLQKELDLLREEKVRLKDILNRKEETLRIMQLHCQEKE 122
TR|A0A2I3SHS9|A0A2I3SHS9_PANTR INEVQQAKKKANKDLGEAWTICEALQKELDSCKRGPVA------LPTETLRILRLHCQEKE 114
SP|Q32LK9|SYCE1_BOVIN             INEVQQAKKKASEELGDARTVWETLQKELDSLSGEKVRLKEILSKKQETLRVLRLHCQDKE 119
TR|F1SCT5|F1SCT5_PIG              INEVQQAKKKASEELGEARTVWETLQKELDSLSGEKVRLKEILSKKQETLRILRLHCQEKE 119
TR|F1PG50|F1PG50_CANLF            INEVQQAKKKASEELGEARTVWEALQKEMDSLSGEKVRLKEILNKKQETLRILRLHCQEKE 104
TR|F7BBC0|F7BBC0_ORNAN            IKDLQRVRKNANEELLKTRAHSEALQRELDELNAEKAHLEEILNQKQETLMMLRLQCEEKQ 115
TR|G3W340|G3W340_SARHA            ISQLQQAQRCTNEELQRNQLKMETLKEELDKLNLEMIQLEETLNMKQGTLLLLQERRREEE 69
TR|H3ALY1|H3ALY1_LATCH            LKQLQQGKNAVDEELCEARKCREALQKELDKLSAESFHLEEIYNKKKETLQLLQFQYKERE 122
TR|K7FPR7|K7FPR7_PELSI            LRKLQRAKQALSQELQDSQARSKELQEELEERCFHPPSWEEICSQKQELLRTLQLRSQETE 74
                                  : .:*: :.  .::*       . *:.*::                  *  :: : .:

SP|Q8N0S2|SYCE1_HUMAN             SEAHRKH--TMLQECKERISALNLQIEEEKNKQRQLRLAFEEQLEDLMGQHKDLWDFHMPE 179
SP|Q9D495|SYCE1_MOUSE             SEAQRKH--SMLQECKERISFLNSQIDKEKAKLRKLRLDFEEHLETLMSQHKDTLEFHKPE 181
TR|A0A2I3SHS9|A0A2I3SHS9_PANTR SEAHRKH--TMLQECKERISALNLQIEEEKNKQRQLRLAFEEQLEDLMGQHKDLWDFHRPE 173
SP|Q32LK9|SYCE1_BOVIN             NEAQRKQ--TMLQECKERISALNSQIEQEKNKQRQLRLDFEEQLEDLMGQYKDLWEFHKPE 178
TR|F1SCT5|F1SCT5_PIG              SEAQRKQ--TMLQECKERISALNSQIEEEKNKQRKLRLDFEEQLEDLMGQHKDLWEFHKPE 178
TR|F1PG50|F1PG50_CANLF            SEAQRKH--TMLQECKERISALNSQIEEEKNKQRQLRLDFEEQLEDLMGQHKDLWKFHGPE 163
TR|F7BBC0|F7BBC0_ORNAN            AEAQRQQ--EVSQGCKQRIEELTSKIQEEKLKQRKQRMEFDQQLEEMMEKHKSLWEFHTSE 174
TR|G3W340|G3W340_SARHA            KKALRQQ--TTSMECLQNVATLNAKIQEEKLRRKKLRKEFEQQLEELMQKHKELMEFHTPQ 128
TR|H3ALY1|H3ALY1_LATCH            NEIKRQL--NHSEGCKQRVEQITSQIQEEKLKRRKQRMEFEMQLEELMEKHKSTWEFHNTE 181
TR|K7FPR7|K7FPR7_PELSI            AEGQRLGCSGLTQERKQHIEELAAKIQEEKLKQRKHRLEFEQLLGELMGEHQSL------E 131
                                   :  *           :.:  :  :*::** : :: *  *:  *  :* :::.       :

SP|Q8N0S2|SYCE1_HUMAN             RLAKEICALDSSKEQLLKEEKLVKATLEDVKHQLCSLCGA----EGPSTLDEGLFLRSQEA 236
SP|Q9D495|SYCE1_MOUSE             HLTKEMCVLDSSKEQLLKEEKLMKVKLEDVRQRLCALGGP----EGSSSLIEGLFLRSHEA 238
TR|A0A2I3SHS9|A0A2I3SHS9_PANTR RLAREICALDSSKEQLLKEEKLVKATLEDMKHQLCSLCGA----EGPSTLDEGLFLRSQEA 230
SP|Q32LK9|SYCE1_BOVIN             RLALEISTLDSGKEQLLKEEKLVEAKLEDVKHRLCSQFGA----KGH-TINEGLFLRSPEA 234
TR|F1SCT5|F1SCT5_PIG              QLALEIDALDSNKEQLLKEEKLVEAKLEDVKHRLCSQFGA----KGCSTITEGLFLRSQEA 235
TR|F1PG50|F1PG50_CANLF            QMAREIDTLDSSKEHLLKEEKLVEAKLEDVKHRLCSQFGA----DGCSTIAEGLFLRSQEA 220
TR|F7BBC0|F7BBC0_ORNAN            SLAREISNIEDSKKHLLNEEKVVQKKIEDIMKQLETLSQ-----PGAAFDSEGLFLRSEEA 230
TR|G3W340|G3W340_SARHA            RLLKEISNLMLTKEQLLEEEKAVQEKLDALEKQIADLPAFMTKEEMMDEGTESVFLHSKEA 189
TR|H3ALY1|H3ALY1_LATCH            SLKKEICNIENAKQQFLSEEKMLQQKLQNLEKEINSLRH-----AGVAFNEEDVFLRSQQA 237
TR|K7FPR7|K7FPR7_PELSI            KLAAEIHSMAESKEHLLSEDRLIQASLAQVEKQLDSLPQ-----ARAALSQERMFLKSQEA 185
                                   :  *:  :   *:::*.*:: :: .:  : :.:                * :**:* :*

SP|Q8N0S2|SYCE1_HUMAN             AATVQLFQEEHRKAEELLAAAAQRHQQLQQKCQQQQQK--RQRLKEELEKHGMQVPAQAQS 295
SP|Q9D495|SYCE1_MOUSE             AAAMQMFKDENKKAEEFLEAAAQQHEQLQQRCHQLQQK--RQRLKEELEKHGVQILAHSTQ 297
TR|A0A2I3SHS9|A0A2I3SHS9_PANTR AATVQLFQEEHRKAEELLAAAAQRHQQLQQKCQQQQQK--RQRLKEELEKHGMQVPAQAQS 289
SP|Q32LK9|SYCE1_BOVIN             AAVVHLFEEENRKAQELLEAAAQRQEQLQQKCQQLQQK--RQRLKEELEKLGVQVLAQAQS 293
TR|F1SCT5|F1SCT5_PIG              AAVVHLFEEENRKAQELLGAAAQRQEQLQQKCQQLQQK--KQRLKEELEKLGVQVPAQSQR 294
TR|F1PG50|F1PG50_CANLF            AAAVHLFEEENRKAQGLLDAATHHHEQLQQKCQQLQQK--RQRLKEELEKLGMQIPVQAQS 279
TR|F7BBC0|F7BBC0_ORNAN            IAAVHLFEEENEKATEFLEAASRHHLELQQKYQR--------------------------- 269
TR|G3W340|G3W340_SARHA            AATMHLFEEENKKAMEFLEAASQKYEMVKQKSL---------------------------- 227
TR|H3ALY1|H3ALY1_LATCH            AVTKQLFEEENSQVKSFLQRASQRHFELQQKCN---------------------------- 275
TR|K7FPR7|K7FPR7_PELSI            STALQLFQQENKSATEHLEAASLRHSELQQKYKRQLFQQENKSATEHLE------------ 239
                                   .. ::*::*: ..    *  *: :   ::*:

SP|Q8N0S2|SYCE1_HUMAN             TQEEEAGPGDVASPKPLKGERPGAA---HQAGPDVLIGQEDTLHPDLSPRGFQEIKELF 351
SP|Q9D495|SYCE1_MOUSE             --NEEDSSWRMASPKPVEVHEETAQDQERPSSRT------------------------- 329
TR|A0A2I3SHS9|A0A2I3SHS9_PANTR TQEEEAGPGDVASPKPLKGERPGAA---LQAGPDVLIGQEDTLHPDLSPRGFQEIKELF 345
SP|Q32LK9|SYCE1_BOVIN             KQEEEAGLGEAANPKPLGVSEEKDQEPSTK----------------------------- 323
TR|F1SCT5|F1SCT5_PIG              KQEEGASPGEAANPKTLGVSEEKDPELPIKQGLMSS----------------------- 330
TR|F1PG50|F1PG50_CANLF            KQEEGAGPGEPANPKLLGVIQEKDPEMPTKEGPMPS----------------------- 315
TR|F7BBC0|F7BBC0_ORNAN            -----------------------------------------------------------
TR|G3W340|G3W340_SARHA            -----------------------------------------------------------
TR|H3ALY1|H3ALY1_LATCH            -----------------------------------------------------------
TR|K7FPR7|K7FPR7_PELSI            -----------------------------------------------------------
```

Fig. S3. Sequence alignment of SYCE1.

Sequence alignment of SYCE1 homologues in vertebrates. Amino acid sequences of *H. sapiens* (human, Q8N0S2), *M. musculus* (mouse, Q9D495), *P. troglodytes* (Chimp,

A0A2I3SHS9), *B. taurus* (Bovine, Q32LK9), *S. scrofa* (Pig, F1SCT5), *C.lupus familiaris* (dog, F1PG50), *O. anatinus* (Platypus, F7BBC0), *S. harrisii* (*Tasmanian devil*, G3W340), *L. chalumnae* (West india coelacanth, H3ALY1) and *P. sinensis* (Chinese turtle, K7FPR7) are derived from the UniProt database. Human and mouse SYCE1 have an overall sequence identity of 65% (78% similarity), with their structural cores exhibiting 74% sequence identity (87% similarity). The residue affected by the POF mutation c.613C>T (p.Gln241*) is conserved between human and mouse, and is indicated in red.

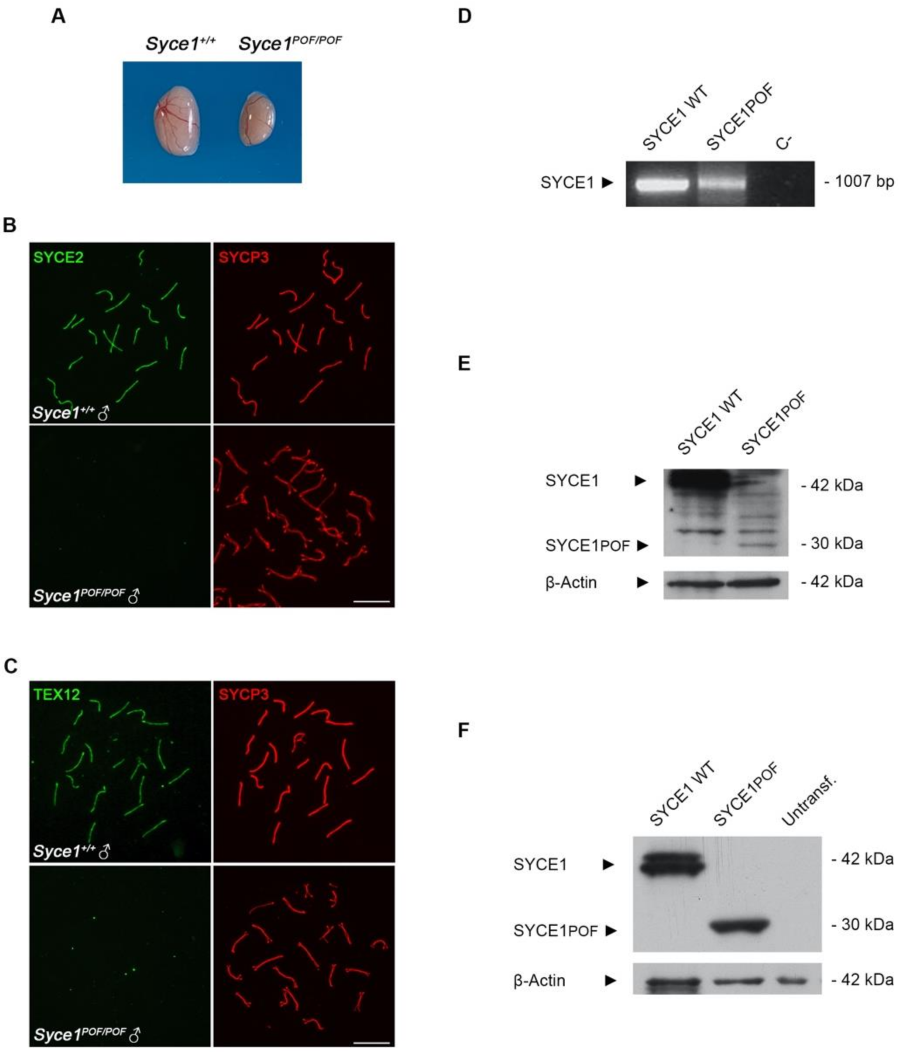

Fig. S4. Characterization of *Syce1*$^{POF/POF}$ male mice whose spermatocytes failed to assemble the CE.

(A) Testis from WT and *Syce1*$^{POF/POF}$ mice showing reduced testis size. **(B-C)** Double immunolabeling of spermatocyte spreads with SYCP3 (red) and the CE proteins SYCE2 and TEX12 (green). *Syce1*$^{POF/POF}$ zygotene-like spermatocytes show absence of both (**B**) SYCE2 and **(C)** TEX12 from the AEs. **(D)** RT-PCR analysis of *Syce1* and *Syce1POF* cDNA from mouse testis. C- lane shows a negative control without cDNA template. Testis RNA was extracted from mice of 13 dpp for the WT and 3 months for the *Syce1POF* in order to have a similar cell content. **(E)** Western blot analysis of protein

extracts from wild type and *Syce1*$^{POF/POF}$ mice testis P13 with a specific antibody against SYCE1. β-Actin was used as loading control. The corresponding bands to SYCE1 and SYCE1POF are indicated in the left of the panel. **(F)** HEK293T cells were transfected with a plasmid encoding mSYCE1 or mSYCEPOF. The whole extracts were analyzed by western blot using rabbit α-SYCE1. Immunodetection of β-actin was used as loading control. The rabbit α-SYCE1 antibody detected both mSYCE1 and mSYCEPOF. Photo credit (A): Laura Gómez-H, Instituto de Biología Celular y Molecular del Cáncer.

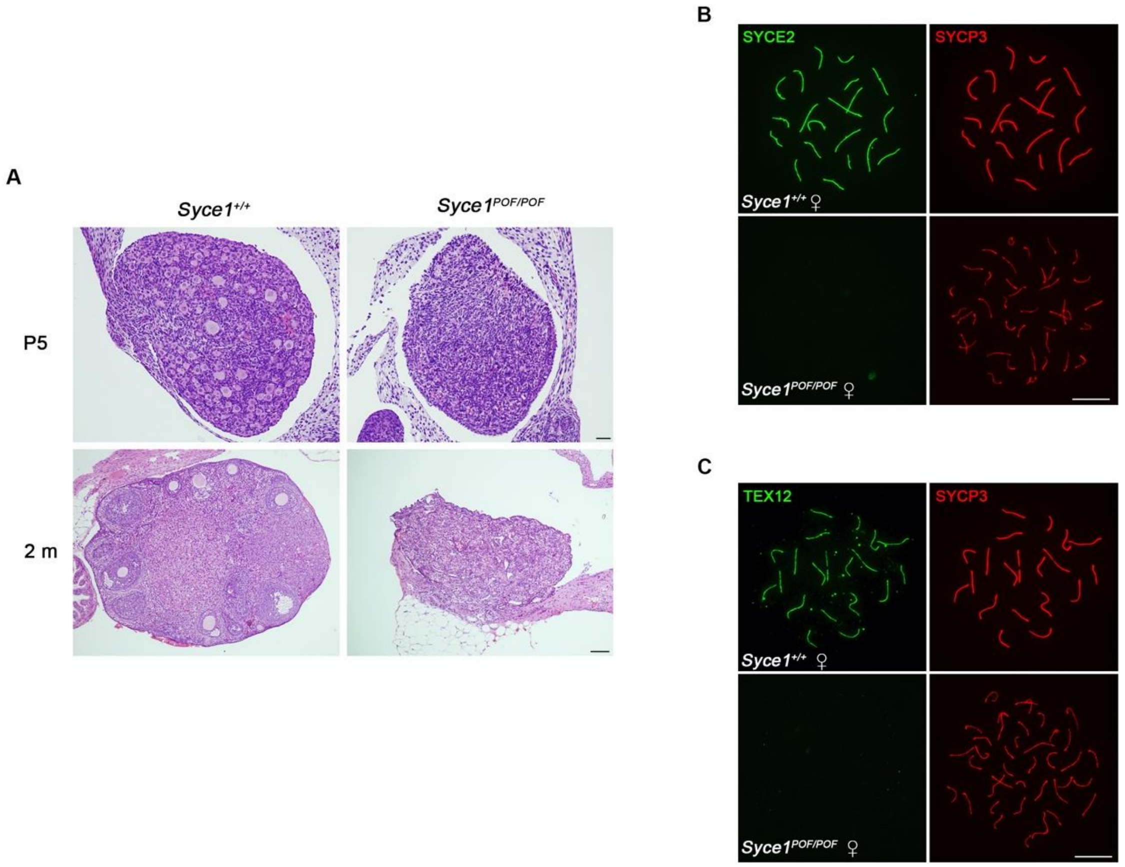

Fig. S5. Syce1POF/POF oocytes are not able to assemble the CE.

(A) Hematoxylin-eosin staining of sections of ovaries from WT and *Syce1*$^{POF/POF}$ female mice showing depletion of follicles at 5 days (P5) and 2 months of age (2m). Scale bars represent 20 μm in P5 and 50 μm at 2m ovaries. **(B-C)** Double immunolabeling of oocyte spreads with SYCP3 (red) and the CE proteins (**B**) SYCE2 and (**C**) TEX12 (green), showing the absence of both CE proteins from the AEs in the *Syce1*$^{POF/POF}$ zygotene-like oocytes. Scale bars represent 10 μm.

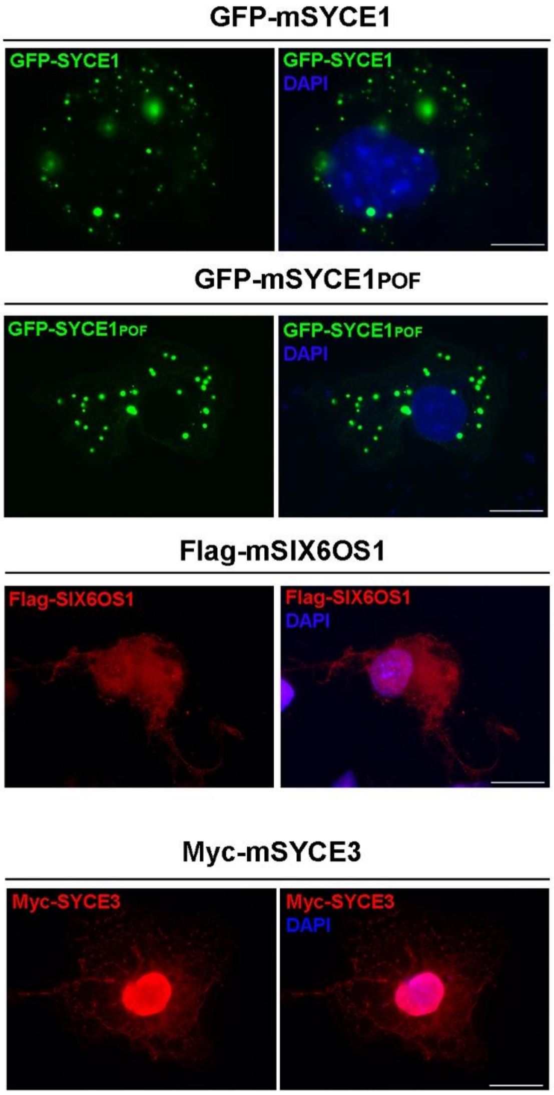

Fig. S6. Cellular localization of CEs overexpressed in COS7 cells.

COS7 cells were transfected with mouse *Syce1*, *Syce1POF*, *Six6os1* and *Syce3* individually as indicated. SYCE1 and SYCE1POF show a speckled pattern in the cytoplasm, while SIX6OS1 has a cytoplasmic distribution. *Syce3* is highly expressed in the nucleus with a widespread low signal in the cytoplasm. Scale bars represent 20 µm.

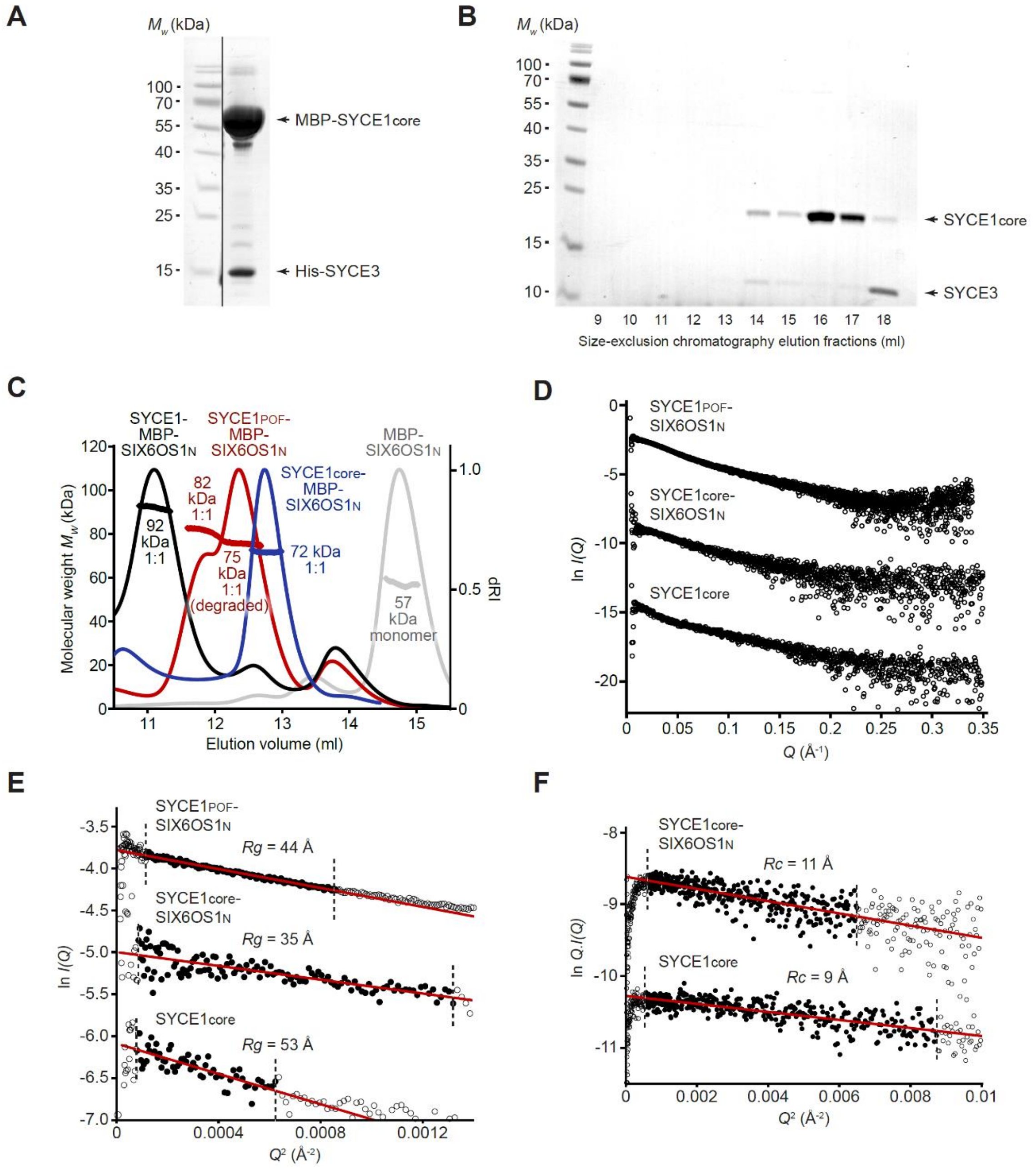

Fig. S7. Biochemical and biophysical analyses of SYCE1 complexes.

(A) Amylose pull-down following co-expression of MBP-SYCE1core and His-SYCE3, demonstrating a direct interaction. (**B**) Size-exclusion chromatography demonstrating the dissociation of SYCE1core and SYCE3 that were purified following co-expression. (**C**) SEC-MALS analysis of His- and MBP-tagged SYCE1-SIX6OS1 complexes. SYCE1core-SIX6OS1N (blue), SYCE1POF-SIX6OS1N (red) and full-length SYCE1-

SIX6OS1N (black) are 1:1 complexes of 72 kDa, 82 kDa (75 kDa for the degradation product complex) and 92 kDa, respectively (theoretical 1:1 – 75 kDa, 83 kDa and 95 kDa). (**D-F**) SEC-SAXS analysis of SYCE1core-SIX6OS1N, SYCE1POF-SIX6OS1N and SYCE1core. (**D**) SEC-SAXS scattering curves. (**E**) SEC-SAXS Guinier analysis to determine the radius of gyration (*Rg*). The linear fits are highlighted in black and are demarcated by dashed lines. The *Q.Rg* values were < 1.3. (**F**) SEC-SAXS Guinier analysis to determine the radius of gyration of the cross-section (*Rc*). The linear fits are highlighted in black and are demarcated by dashed lines. The *Q.Rc* values were < 1.3.

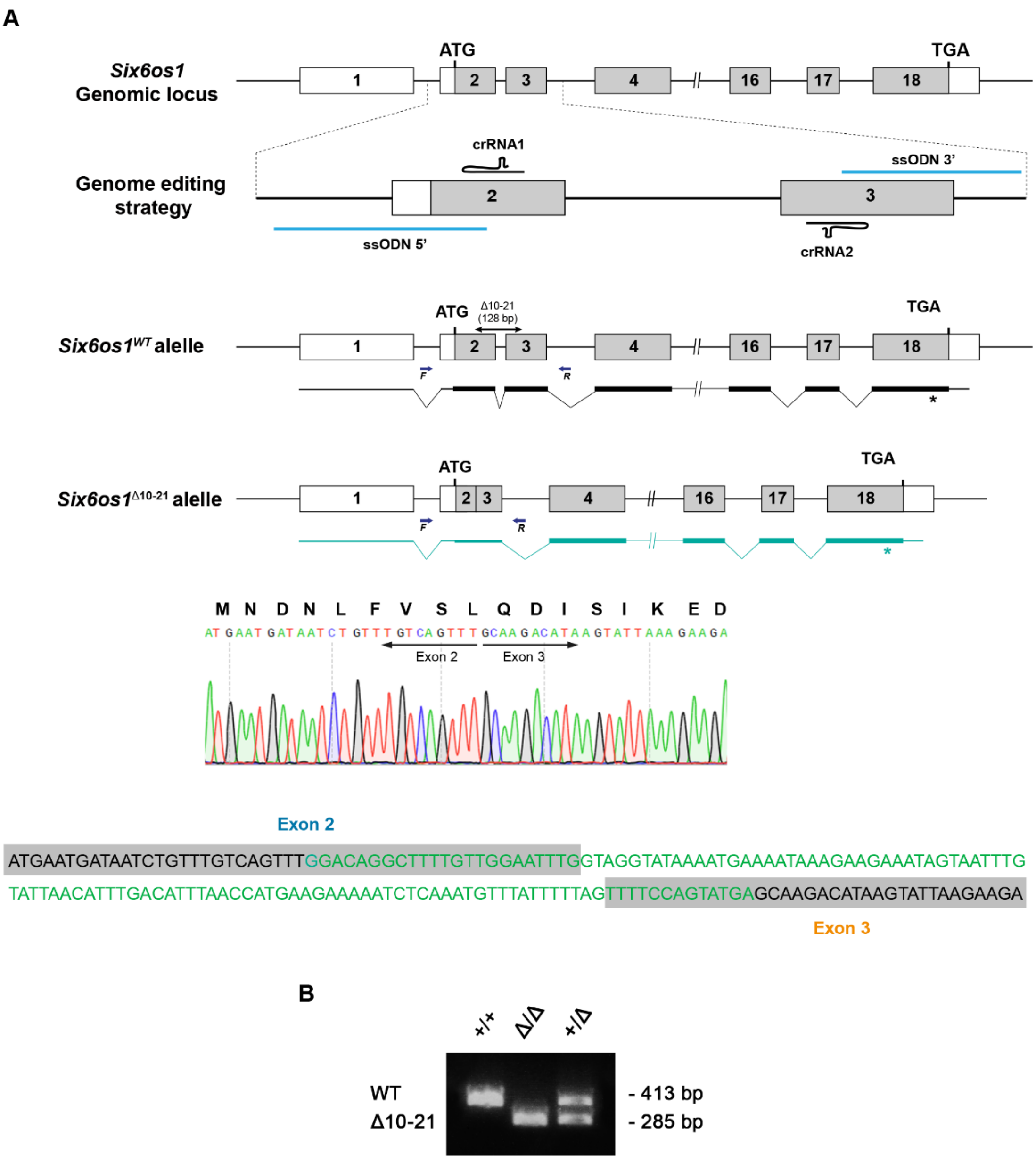

Fig. S8. Generation of *Six6os1*$^{Δ10-21/Δ10-21}$ mutant mice.

(A) Schematic representation of the genome editing strategy for the generation of the *Six6os1* Δ10-21 mutant mouse model showing the crRNAs located on exons 2 and 3. The ssODN comprises the ssODN 5' and the ssODN 3' sequences, targeting the 5' of exon 2 and the 3' of exon 3 respectively (see methods). The corresponding coding exons (grey boxes) and non-coding exons (open boxes) are represented. Thin (non-coding) and thick (coding sequences) lines under exons represent the expected transcript

derived from wild-type (black) and *Six6os1* Δ10-21 edited allele (green). ATG, initiation codon; TGA and *, stop codon. Primers for PCR genotyping (F and R) are represented by arrows. The nucleotide sequence of the edited region shows the deletion of 128 bp derived from PCR amplification of DNA from the *Six6os1*$^{\Delta 10\text{-}21/\Delta 10\text{-}21}$ (named in the figures *Six6os1*$^{\Delta/\Delta}$), in addition to the corresponding amino-acids sequence. The sequence of the edited region of *Six6os1* is indicated below labelling the deleted region in green letters. **(B)** PCR analysis of genomic DNA from three littermate progeny of *Six6os1*$^{\Delta 10\text{-}21/WT}$ crosses. The PCR amplification with primers F and R revealed 413 and 285 bp fragments for wild-type and edited alleles respectively. Wild-type (+/+), Δ10-21 homozygous (Δ/Δ) and heterozygous (Δ/+) animals.

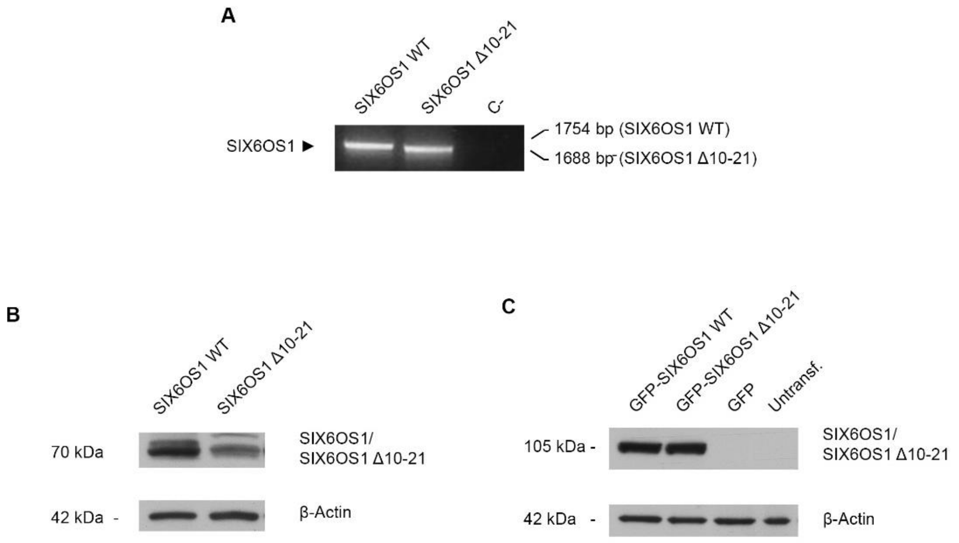

Fig. S9. Analysis of the expression of *Six6os1*$^{\Delta 10\text{-}21}$

(A) RT-PCR analysis of *Six6os1* and *Six6os1*$^{\Delta 10\text{-}21}$ cDNA from mouse testis. C- lane shows a negative control without cDNA template. RNA was extracted from mice of 13 dpp for the WT and 3 months for the *Six6os1*$^{\Delta 10\text{-}21}$ in order to have a similar cell content. **(B)** Western blot analysis of protein testis extracts from *Six6os1*$^{\Delta 10\text{-}21/\Delta 10\text{-}21}$ and wild type adult mice with a specific antibody against SIX6OS1. β-Actin was used as loading control. The corresponding bands to SIX6OS1 and SIX6OS1 Δ10-21 are indicated in the left of the panel. **(C)** HEK293T cells were transfected with a plasmid encoding GFP-mSIX6OS1 or GFP-mSIX6OS1 Δ10-21. The whole extracts were analyzed by western blot using rabbit α-SIX6OS1. Immunodetection of β-actin was used as loading control.

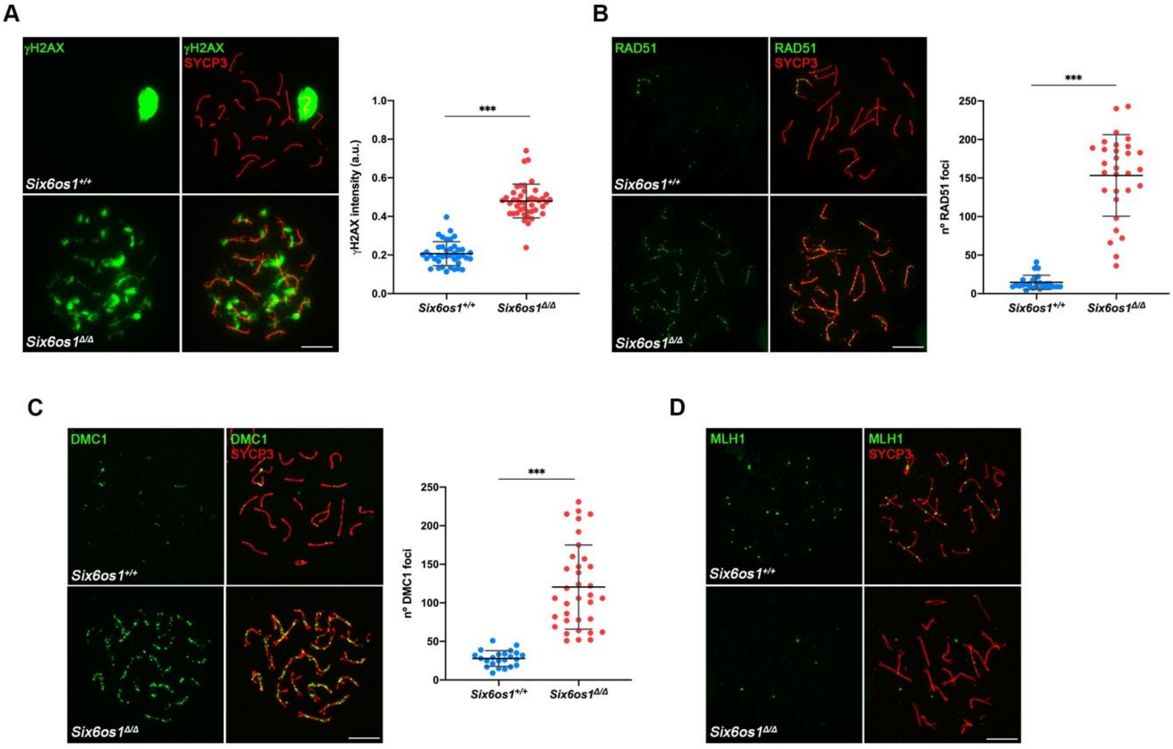

Fig. S10. DSBs are generated but defectively repaired in *Six6os1*$^{\Delta10\text{-}21/\Delta10\text{-}21}$ spermatocytes.

(A) Double immunofluorescence of γ-H2AX (green) and SYCP3 (red) in spermatocyte spreads from WT and *Six6os1*$^{\Delta/\Delta}$ mice. In WT pachytenes γ-H2AX intensely labels the chromatin of the sex bivalent, while in the *Six6os1*$^{\Delta/\Delta}$ arrested zygotene-like γ-H2AX labeling remains in the chromatin. **(B-C)** Double immunolabeling of **(B)** RAD51 or **(C)** DMC1 (green) and SYCP3 (red). Both RAD51 and DMC1 remain associated to the AEs in the *Six6os1*$^{\Delta/\Delta}$ zygotene-like spermatocytes, showing increased number of foci than the WT. **(D)** Double immunolabeling of MLH1 (green) and SYCP3 (red). MLH1 is absent from the AEs in the *Six6os1*$^{\Delta/\Delta}$ arrested spermatocytes. Scale bars represent 10 µm. Plots under the image panels represent the quantification of fluorescence intensity **(A)** or number of foci **(B-C)** from WT and zygotene-like arrested spermatocytes. Welch´s *t*-test analysis: * p<0.01, **p<0.001, ***p<0.0001.

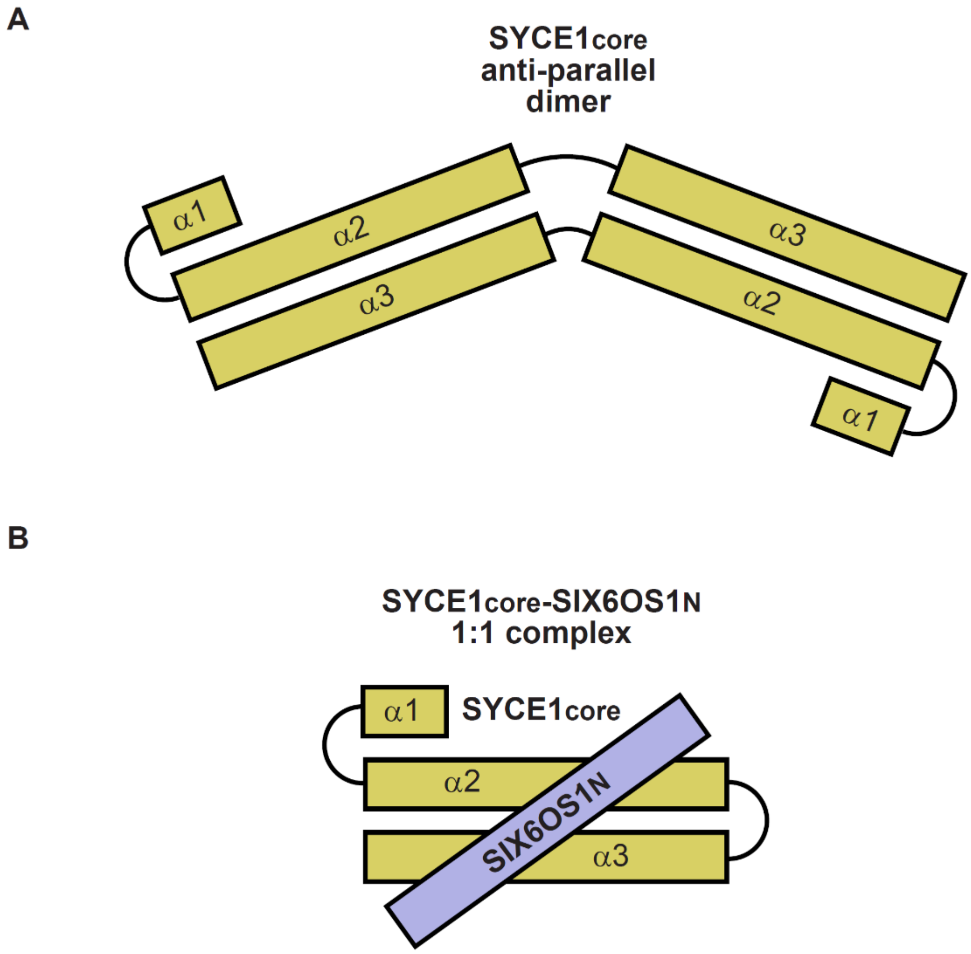

Fig. S11. Structural models of SYCE1core and SYCE1core-SIX6OS1N.

(**A,B**) Theoretical structural models of (**A**) the SYCE1core anti-parallel dimer and (**B**) the SYCE1core-SIX6OS1N 1:1 complex. The SYCE1core helices labelled α1-3 correspond to approximate amino-acid boundaries of 25-50, 52-113 and 118-179, respectively. The models presented are compatible with the biophysical data presented herein and our previously reported structure of SYCE1core (*28*). We propose that SIX6OS1N-binding may occur through a single SYCE1core chain undergoing helix-turn-loop formation between helices α2 and α2, to create a structure that resembles one half of the SYCE1core dimer and interacts with SIX6OS1N as a 1:1 complex.

A

	Condition					*β-Actin*						
	Ct_1	Ct_2	Ct_3	Ct_{mean}	2^{-Ct}	Ct_1	Ct_2	Ct_3	Ct_{mean}	2^{-Ct}	Normalized 2^{-Ct}	WT/POF Ratio
Syce1$^{+/+}$	25.09	25.58	24.60	**25.09**	$2.80 \cdot 10^{-8}$	16.55	16.65	16.87	**16.69**	$9.46 \cdot 10^{-6}$	**2.96**$\cdot 10^{-3}$	**46.96**
Syce1$^{POF/POF}$	29.27	29.20	30.00	**29.49**	$1.33 \cdot 10^{-9}$	15.17	15.40	16.04	**15.54**	$0.10 \cdot 10^{-5}$	**6.30**$\cdot 10^{-5}$	

B

	Condition				*β-Actin*					
	Ct_1	Ct_2	Ct_{mean}	2^{-Ct}	Ct_1	Ct_2	Ct_{mean}	2^{-Ct}	Normalized 2^{-Ct}	WT/Δ Ratio
Six6os1$^{+/+}$	24.96	23.02	**23.99**	$6.00 \cdot 10^{-8}$	11.97	15.75	**13.86**	$6.73 \cdot 10^{-5}$	**8.92**$\cdot 10^{-4}$	**0.29**
Six6os1$^{\Delta/\Delta}$	21.28	22.08	**21.68**	$2.98 \cdot 10^{-7}$	13.09	13.58	**13.34**	$9.68 \cdot 10^{-5}$	**3.08**$\cdot 10^{-3}$	

Table S1. Analysis of the relative expression of *Syce1POF* and *Six6os1$^{\Delta 10\text{-}21}$* in testis from mutant mice.

(**A**) Relative transcription of *Syce1 and Syce1POF* mRNA by RT-qPCR in testis from WT and *Syce1$^{POF/POF}$* mice. (**B**) Relative transcription of *Six6os1 and Six6os1$^{\Delta 10\text{-}21}$* mRNA by RT-qPCR in testis from WT and *Six6os1$^{\Delta 10\text{-}21/\ \Delta 10\text{-}21}$* mice. *β-Actin* transcription was used to normalize the expression in both (**A**) and (**B**). RNA was extracted from testis at 13 dpp for the WT and 3 months of age for the mutant mice in order to have similar germ cell content, showing zygotenes as the most developed cell type.
